# Species conservation profiles of cave-adapted terrestrial isopods from Portugal

**DOI:** 10.3897/BDJ.10.e78796

**Published:** 2022-02-28

**Authors:** Ana Sofia P. S. Reboleira, Rita P. Eusébio, Stefano Taiti

**Affiliations:** 1 Centre for Ecology, Evolution and Environmental Changes (cE3c), and Departamento de Biologia Animal, Faculdade de Ciências, University of Lisbon, Lisbon, Portugal Centre for Ecology, Evolution and Environmental Changes (cE3c), and Departamento de Biologia Animal, Faculdade de Ciências, University of Lisbon Lisbon Portugal; 2 Natural History Museum of Denmark, University of Copenhagen, Copenhagen, Denmark Natural History Museum of Denmark, University of Copenhagen Copenhagen Denmark; 3 Istituto di Ricerca sugli Ecosistemi Terrestri, Florence, Italy Istituto di Ricerca sugli Ecosistemi Terrestri Florence Italy

**Keywords:** Oniscidea, subterranean habitats, troglobiont, cavernicolous, Iberian Peninsula, conservation, rocky habitats

## Abstract

**Background:**

Terrestrial isopods (Oniscidea) are the most diverse group of troglobionts in caves of continental Portugal. They occur in all karst regions of Portugal, play a major role in decomposition of organic matter in caves and may act as umbrella species for the conservation of all other cave-adapted invertebrates.

**New information:**

We present the IUCN Red List profiles for the cave-adapted terrestrial isopods from continental Portugal, based on recent distribution data from caves.

## Introduction

Cave-adapted fauna have a high and global conservationist interest (Mammola et al. 2019). Cave-adapted communities have typically high endemicity patterns and are composed of a small number of species with small populations; hence, specific conservation measures should be defined and established regionally (Reboleira et al. 2011, Reboleira et al. 2013a, Reboleira and Eusébio 2021).

Terrestrial isopods (Oniscidea) evolved to be the only truly terrestrial crustaceans (Hornung 2011). They play a vital role on decomposition of organic matter in the soil (Hassall et al. 1987), contributing to the biogeochemical cycles on Earth (Ravn et al. 2020). Therefore, terrestrial isopods can be used as bioindicators of soil ecosystem health (van Gestel et al. 2018).

In subterranean ecosystems, terrestrial isopods can be seen as sentinel organisms, as they are detritivorous, basal to the cave food webs, source of food for many predators, are generally associated with stable and undisturbed parts of caves and are the most diversified group (Campos-Filho et al. 2014, Reboleira et al. 2015).

Portugal is a hotspot of subterranean biodiversity and, amongst all cave-adapted species, terrestrial isopods are the most diverse, represented in mainland by 15 troglobiont species (Reboleira 2012, Reboleira et al. 2015, Vandel 1946). Trichoniscidae is the most diverse cave-adapted isopod family, with 12 troglobiotic species, followed by Armadillidiidae, with two species and Styloniscidae and Porcellionidae, with one species each (Reboleira et al. 2015).

Cave-adapted species of mainland Portugal lack specific protective legislation and Red List profiling (Reboleira and Eusébio 2021). In this paper, we created IUCN Red List profiles for 15 species of cave-adapted isopods from continental Portugal.

## Material and Methods

Over the last 15 years, the caves of continental Portugal have been extensively sampled by direct search and baited pitfall traps. The specimens were sorted and identified to species level through dissection, microscopy, comparison with collection specimens and bibliography (Vandel 1946, Reboleira et al. 2015).

Extent of occurence (EOO) and area of occupancy (AOO) were calculated using the Geospatial Conservation Assessment Tool (GeoCAT) with an approximation to the standard IUCN 2 km × 2 km cells (4 km^2^). The maps were generated in the open source software QGIS 3.14.16, with the layer of the natural protected areas of Portugal (ICNF 2020).

Threats were identified *in situ*, complemented by a literature survey and spatial analysis software. The type of habitat classification, threats and conservation actions were assigned, based on the IUCN Red List database.

## Species Conservation Profiles

### Trichoniscoides bellesi

#### Species information

Scientific name: Trichoniscoidesbellesi

Species authority: Reboleira & Taiti, 2015

Kingdom: Animalia

Phylum: Arthropoda

Class: Malacostraca

Order: Isopoda

Family: Trichoniscidae

Taxonomic notes: *Trichoniscoidesbellesi* displays troglomorphisms, like blindness and depigmentation. It can be easily distinguished from other species of the genus because the exopod of the first pleopod of the male has a broadly rounded outer margin and two equal distal lobes and the endopod of the second pleopod has a distal article thickset for ⅔ of its length, with a narrow terminal part (Reboleira et al. 2015).

Region for assessment: Europe

#### Editor & Reviewers

##### Reviewers

Reviewers: Reviewers

##### Editor

Editor: Editor

#### Reviewers

Reviewers: Reviewers

#### Editor

Editor: Editor

#### Geographic range

Biogeographic realm: Palearctic

Countries: Portugal

Map of records (Google Earth): Suppl. materials 1, 2

Basis of EOO and AOO: Known habitat extent

Basis (narrative): Both the extent of occurrence (EOO) and area of occupancy (AOO) are 4 km^2^.

Min Elevation/Depth (m): 380

Range description: *Trichoniscoidesbellesi* is known from Algar do Javali Cave in the Montejunto karst massif (Reboleira et al. 2015).

#### New occurrences

#### Extent of occurrence

EOO (km2): 4

Trend: Unknown

Causes ceased?: Unknown

Causes understood?: Unknown

Causes reversible?: Unknown

Extreme fluctuations?: Unknown

#### Area of occupancy

Trend: Unknown

Causes ceased?: Unknown

Causes understood?: Unknown

Causes reversible?: Unknown

Extreme fluctuations?: Unknown

AOO (km2): 4

#### Locations

Number of locations: 1

Justification for number of locations: *Trichoniscoidesbellesi* occurs in a single cave (Reboleira et al. 2015).

Trend: Stable

Justification for trend: Algar do Javali Cave is the only known location for this species; therefore, the trend in number of locations is stable.

Extreme fluctuations?: Unknown

#### Population

Number of individuals: Unknown

Trend: Unknown

Causes ceased?: Unknown

Causes understood?: Unknown

Causes reversible?: Unknown

Extreme fluctuations?: Unknown

Population Information (Narrative): A total of three specimens have been collected in the type locality (Reboleira et al. 2015).

#### Subpopulations

Trend: Unknown

Extreme fluctuations?: Unknown

Severe fragmentation?: Unknown

#### Habitat

System: Terrestrial

Habitat specialist: Yes

Habitat (narrative): *Trichoniscoidesbellesi* was found in the deepest and most thermally insulated parts of the cave, at 10 m depth (Reboleira et al. 2015).

Trend in extent, area or quality?: Decline (inferred)

##### Habitat

Habitat importance: Major Importance

Habitats: 7.1. Caves and Subterranean Habitats (non-aquatic) - Caves

#### Habitat

Habitat importance: Major Importance

Habitats: 7.1. Caves and Subterranean Habitats (non-aquatic) - Caves

#### Ecology

Size: 1.7 mm (male), 1.8 mm (female)

Generation length (yr): 1

Dependency of single sp?: Unknown

Ecology and traits (narrative): *Trichoniscoidesbellesi* is a blind and depigmented troglobiont species (Reboleira et al. 2015). It shares habitat with other endemic cave-adapted species, such as the pseudoscorpions *Occidenchthoniuscardosoi* (Zaragoza, 2012) (Zaragoza and Reboleira 2018) and *Roncocreagrisoccidentalis* Zaragoza & Reboleira, 2013 (Reboleira et al. 2013b); an undescribed species of terrestrial isopod *Paraschizidium* Verhoeff, 1919; the beetle *Trechustatai* Reboleira & Ortuño, 2010 (Reboleira et al. 2010a, Reboleira et al. 2015) and a new species of the pselaphid beetle *Tychobythinus* Ganglbauer, 1896. Algar do Javali Cave has a mean annual temperature of 14.4°C and an amplitude of 1.1°C at the sediment level, measured hourly during 2010.

#### Threats

Justification for threats: The cave entrance is surrounded by *Eucalyptus* intensive plantations, which substituted the original native vegetation and is located 50 m from a road, 1.6 km from a quarry and 2.9 km from the closest village.

##### Threats

Threat type: Ongoing

Threats: 1.1. Residential & commercial development - Housing & urban areas2.2. Agriculture & aquaculture - Wood & pulp plantations3.2. Energy production & mining - Mining & quarrying4. Transportation & service corridors

#### Threats

Threat type: Ongoing

Threats: 1.1. Residential & commercial development - Housing & urban areas2.2. Agriculture & aquaculture - Wood & pulp plantations3.2. Energy production & mining - Mining & quarrying4. Transportation & service corridors

#### Conservation

Justification for conservation actions: Even though this cave is protected under legislation by the “Rede Natura 2000” (Directive 1992, ICNB 2000), this rare and single-cave endemic terrestrial isopod species lacks specific protection measures. The development of a conservation plan for this cave area is crucial to ensure its environmental sustainability and the survival of the species.

##### Conservation actions

Conservation action type: Needed

Conservation actions: 1.1. Land/water protection - Site/area protection1.2. Land/water protection - Resource & habitat protection4. Education & awareness5.1.3. Law & policy - Legislation - Sub-national level

#### Conservation actions

Conservation action type: Needed

Conservation actions: 1.1. Land/water protection - Site/area protection1.2. Land/water protection - Resource & habitat protection4. Education & awareness5.1.3. Law & policy - Legislation - Sub-national level

#### Other

##### Use and trade

##### Ecosystem services

#### Use and trade

#### Ecosystem services

#### Viability analysis

### Trichoniscoides broteroi

#### Species information

Scientific name: Trichoniscoidesbroteroi

Species authority: Vandel, 1946

Kingdom: Animalia

Phylum: Arthropoda

Class: Malacostraca

Order: Isopoda

Family: Trichoniscidae

Region for assessment: Europe

#### Editor & Reviewers

##### Reviewers

Reviewers: Reviewers

##### Editor

Editor: Editor

#### Reviewers

Reviewers: Reviewers

#### Editor

Editor: Editor

#### Geographic range

Biogeographic realm: Palearctic

Countries: Portugal

Map of records (Google Earth): Suppl. materials 1, 3

Basis of EOO and AOO: Known habitat extent

Basis (narrative): The extent of occurrence (EOO) and area of occupancy (AOO) are both 4 km^2^.

Min Elevation/Depth (m): 100

Range description: *Trichoniscoidesbroteroi* is a troglobiont isopod known from Alqueves Cave, located in the Sicó karst area (Reboleira et al. 2015). This cave forms a gallery of about 6 m long (Grilo et al. 2016).

#### New occurrences

#### Extent of occurrence

EOO (km2): 4

Trend: Unknown

Causes ceased?: Unknown

Causes understood?: Unknown

Causes reversible?: Unknown

Extreme fluctuations?: Unknown

#### Area of occupancy

Trend: Unknown

Causes ceased?: Unknown

Causes understood?: Unknown

Causes reversible?: Unknown

Extreme fluctuations?: Unknown

AOO (km2): 4

#### Locations

Number of locations: 1

Justification for number of locations: *Trichoniscoidesbroteroi* is known from a single cave (Reboleira et al. 2015).

Trend: Stable

Justification for trend: Alqueves Cave is the only known location for this species; therefore, the trend in number of locations is stable.

Extreme fluctuations?: Unknown

#### Population

Number of individuals: Unknown

Trend: Unknown

Causes ceased?: Unknown

Causes understood?: Unknown

Causes reversible?: Unknown

Extreme fluctuations?: Unknown

#### Subpopulations

Trend: Unknown

Extreme fluctuations?: Unknown

Severe fragmentation?: Unknown

#### Habitat

System: Terrestrial

Habitat specialist: Yes

Habitat (narrative): Alqueves Cave is considered a pre- and proto-historic site. It is a natural funerary cave as bone fragments and other human artifacts were there found, testifying to its human occupation at the end of the Neolithic period, beginning of the Chalcolithic (Grilo et al. 2016). This cave has a corridor shape, 5–6 m long and it is divided into two galleries, with an extremely clayey floor, with a depression in its central part that, in winter, forms a small lake (Grilo et al. 2016). The cave is currently closed by the Municipality and the surrounding area is fully urbanised (Reboleira et al. 2015).

Trend in extent, area or quality?: Decline (inferred)

##### Habitat

Habitat importance: Major Importance

Habitats: 7.1. Caves and Subterranean Habitats (non-aquatic) - Caves

#### Habitat

Habitat importance: Major Importance

Habitats: 7.1. Caves and Subterranean Habitats (non-aquatic) - Caves

#### Ecology

Size: 4 mm (male), 4.5 mm (female)

Generation length (yr): 1

Dependency of single sp?: Unknown

Ecology and traits (narrative): *Trichoniscoidesbroteroi* is a blind and depigmented troglobiont species and a single cave endemic (Reboleira et al. 2015). It shares habitat with another cave-adapted terrestrial isopod, the species *Porcelliocavernicolus* (Vandel 1946).

#### Threats

Justification for threats: The entrance to the cave is located within a fully urbanised area, more specifically in the middle of a roundabout (Grilo et al. 2016, Reboleira et al. 2015). This cave is located 1.3 km from the Mondego River and 2 km from the city centre of Coimbra.

##### Threats

Threat type: Ongoing

Threats: 1.1. Residential & commercial development - Housing & urban areas1.3. Residential & commercial development - Tourism & recreation areas4. Transportation & service corridors9.1. Pollution - Domestic & urban waste water9.5. Pollution - Air-borne pollutants

#### Threats

Threat type: Ongoing

Threats: 1.1. Residential & commercial development - Housing & urban areas1.3. Residential & commercial development - Tourism & recreation areas4. Transportation & service corridors9.1. Pollution - Domestic & urban waste water9.5. Pollution - Air-borne pollutants

#### Conservation

Justification for conservation actions: This cave was previously excavated for archaeological studies where bone fragments, chipped stone material, ceramic fragments, quartzite blades and bone pins were found (Grilo et al. 2016). It is also the only location for *Trichoniscoidesbroteroi* (Reboleira et al. 2015). Despite its archaeological and ecological importance and the severe threats it faces, this cave is not adequately protected for biodiversity; therefore, conservation of this habitat is crucial. Measures should also be taken to prevent infiltration of wastewaters and urban residues into the subterranean habitat.

##### Conservation actions

Conservation action type: Needed

Conservation actions: 1.1. Land/water protection - Site/area protection2.1. Land/water management - Site/area management2.3. Land/water management - Habitat & natural process restoration4. Education & awareness5.1.3. Law & policy - Legislation - Sub-national level

#### Conservation actions

Conservation action type: Needed

Conservation actions: 1.1. Land/water protection - Site/area protection2.1. Land/water management - Site/area management2.3. Land/water management - Habitat & natural process restoration4. Education & awareness5.1.3. Law & policy - Legislation - Sub-national level

#### Other

##### Use and trade

##### Ecosystem services

#### Use and trade

#### Ecosystem services

#### Viability analysis

### Trichoniscoides meridionalis

#### Species information

Scientific name: Trichoniscoidesmeridionalis

Species authority: Vandel, 1946

Kingdom: Animalia

Phylum: Arthropoda

Class: Malacostraca

Order: Isopoda

Family: Trichoniscidae

Region for assessment: Europe

#### Editor & Reviewers

##### Reviewers

Reviewers: Reviewers

##### Editor

Editor: Editor

#### Reviewers

Reviewers: Reviewers

#### Editor

Editor: Editor

#### Geographic range

Biogeographic realm: Palearctic

Countries: Portugal

Map of records (Google Earth): Suppl. materials 1, 4

Basis of EOO and AOO: Known habitat extent

Basis (narrative): The extent of occurrence (EOO) is 217.7 km^2^ and the area of occupancy (AOO) is 40 km^2^.

Min Elevation/Depth (m): 95

Max Elevation/Depth (m): 485

Range description: *Trichoniscoidesmeridionalis* is recorded from 10 caves distributed along the Estremenho karst massif: Algar do Vale da Pena, Algar do Zé de Braga, Alcobertas, Lapa da Chã de Cima, Moinhos Velhos, Almonda, Papagaio, Algar do Burro, Algar do Pena and Algar do Ladoeiro (Vandel 1946, Reboleira et al. 2015, Reboleira and Enghoff 2018).

#### New occurrences

#### Extent of occurrence

EOO (km2): 217.7

Trend: Unknown

Causes ceased?: Unknown

Causes understood?: Unknown

Causes reversible?: Unknown

Extreme fluctuations?: Unknown

#### Area of occupancy

Trend: Unknown

Causes ceased?: Unknown

Causes understood?: Unknown

Causes reversible?: Unknown

Extreme fluctuations?: Unknown

AOO (km2): 40

#### Locations

Number of locations: 10

Justification for number of locations: *Trichoniscoidesmeridionalis* occurs in ten caves (Reboleira et al. 2015, Reboleira and Enghoff 2018).

Trend: Unknown

Extreme fluctuations?: Unknown

#### Population

Number of individuals: Unknown

Trend: Unknown

Causes ceased?: Unknown

Causes understood?: Unknown

Causes reversible?: Unknown

Extreme fluctuations?: Unknown

Population Information (Narrative): A total of 43 specimens have been collected: six in Moinhos Velhos cave, five in Algar do Vale do Pena, five in Algar do Burro, one in Algar do Zé de Braga, fifteen in Algar do Ladoeiro, ten in Almonda and one in Papagaio Caves (Reboleira et al. 2015).

#### Subpopulations

Number of subpopulations: 10

Trend: Decline (inferred)

Extreme fluctuations?: Unknown

Severe fragmentation?: Unknown

#### Habitat

System: Terrestrial

Habitat specialist: Yes

Habitat (narrative): The caves are located at an altitude ranging from 95 to 485 m a.s.l. (Reboleira et al. 2015). Amonda Cave represents the easternmost locality for the species' distribution, while Algar do Vale da Pena is the westernmost. Almonda is the largest cave in Portugal, with more than 10 km of mapped galleries (Thomas 1991).

Trend in extent, area or quality?: Decline (inferred)

##### Habitat

Habitat importance: Major Importance

Habitats: 7.1. Caves and Subterranean Habitats (non-aquatic) - Caves

#### Habitat

Habitat importance: Major Importance

Habitats: 7.1. Caves and Subterranean Habitats (non-aquatic) - Caves

#### Ecology

Size: 2 mm (male), 2.5 mm (female)

Generation length (yr): 1

Dependency of single sp?: Unknown

Ecology and traits (narrative): *Trichoniscoidesmeridionalis* is a blind, depigmented troglobiont that is adapted to life in the underground (Vandel 1946, Reboleira et al. 2015). This species can be found both close to the entrance and at the deepest parts of caves, usually associated with decomposing organic matter (Reboleira 2007, Reboleira et al. 2009, Reboleira et al. 2015).

#### Threats

Justification for threats: Almonda Cave is located 50 m from a factory that extracts water from a subterranean river and 420 m from a village, which has many agricultural fields. Algar do Ladoeiro's Cave entrance is 840 m from the closest urbanisation. The Moinhos Velhos Cave is the largest show cave of Portugal with around 140,000 visitors per year. Alcobertas Cave has also been subject to structural alterations with the intention to transform it into a show cave during the last century and it is exploited for tourism by a local association (Reboleira et al. 2009). The subterranean stream in Moinhos Velhos Cave has high input of sewage from the surface and is located below the village of Mira d'Aire; therefore, this cave is extremely contaminated by surface pollutants (Reboleira 2007), its entrance is located in the village centre and, since the 1960s, has had much infrastructure built for touristic exploitation, with a 300 m long show cave section (Reboleira et al. 2015). Alcobertas Cave has been intensively exploited for touristic activities since the 70s, where a second entrance has been opened, drastically changing the climatic conditions (Reboleira 2007, Reboleira et al. 2009). This cave is located 640 m from a field of energy windmills, 1 km from a quarry, 850 m from agricultural lands and 690 m from the nearest village. Algar do Vale da Pena is located in an abandoned quarry, 700 m from the closest village. Papagaio Cave is located 120 m from a quarry and 1 km from a major highway, A1. Algar do Burro is located 500 m from a quarry, 560 m from the A1 highway and 600 m from the closest village. Lapa da Chã de Cima is located 500 m from a quarry. Algar do Zé de Braga is located below intensive agricultural olive production, which is threatened by the use of pesticides at the surface, known to percolate into the underground and cause pernicious effects on subterranean biota (Castaño-Sánchez et al. 2020, Reboleira et al. 2013a).

##### Threats

Threat type: Ongoing

Threats: 1.1. Residential & commercial development - Housing & urban areas1.2. Residential & commercial development - Commercial & industrial areas2.1. Agriculture & aquaculture - Annual & perennial non-timber crops3.2. Energy production & mining - Mining & quarrying3.3. Energy production & mining - Renewable energy6.1. Human intrusions & disturbance - Recreational activities9.1.2. Pollution - Domestic & urban waste water - Run-off

#### Threats

Threat type: Ongoing

Threats: 1.1. Residential & commercial development - Housing & urban areas1.2. Residential & commercial development - Commercial & industrial areas2.1. Agriculture & aquaculture - Annual & perennial non-timber crops3.2. Energy production & mining - Mining & quarrying3.3. Energy production & mining - Renewable energy6.1. Human intrusions & disturbance - Recreational activities9.1.2. Pollution - Domestic & urban waste water - Run-off

#### Conservation

Justification for conservation actions: Almonda Cave is classified, since 1993, as a Property of Public Interest (IIP) and protected due to its archaeological heritage (Hoffmann et al. 2013). The archaeological arguments are, however, inappropriate for cave-adapted fauna protection, so it is urgent to set protective measures directed at the cave fauna. Of the ten caves, only six are protected under legislation by the “Rede Natura 2000” (Directive 1992, ICNB 2000). Measures need to be established to protect the habitats and locations that are affected by human activities, wastewater infiltration and pollution and to implement protection on the locations neglected under the current legislation.

##### Conservation actions

Conservation action type: Needed

Conservation actions: 1.1. Land/water protection - Site/area protection1.2. Land/water protection - Resource & habitat protection2.1. Land/water management - Site/area management4. Education & awareness5.1.3. Law & policy - Legislation - Sub-national level

#### Conservation actions

Conservation action type: Needed

Conservation actions: 1.1. Land/water protection - Site/area protection1.2. Land/water protection - Resource & habitat protection2.1. Land/water management - Site/area management4. Education & awareness5.1.3. Law & policy - Legislation - Sub-national level

#### Other

##### Use and trade

##### Ecosystem services

#### Use and trade

#### Ecosystem services

#### Viability analysis

### Trichoniscoides ouremensis

#### Species information

Scientific name: Trichoniscoidesouremensis

Species authority: Vandel, 1946

Kingdom: Animalia

Phylum: Arthropoda

Class: Malacostraca

Order: Isopoda

Family: Trichoniscidae

Region for assessment: Europe

#### Editor & Reviewers

##### Reviewers

Reviewers: Reviewers

##### Editor

Editor: Editor

#### Reviewers

Reviewers: Reviewers

#### Editor

Editor: Editor

#### Geographic range

Biogeographic realm: Palearctic

Countries: Portugal

Map of records (Google Earth): Suppl. materials 1, 5

Basis of EOO and AOO: Known habitat extent

Basis (narrative): Both the extent of occurrence (EOO) and area of occupancy (AOO) are 4 km^2^.

Range description: *Trichoniscoidesouremensis* is only recorded from Lapa da Salgada Cave, located in the Fátima Plateau, in the eastern part of the Estremenho karst massif (Vandel 1946, Reboleira et al. 2015).

#### New occurrences

#### Extent of occurrence

EOO (km2): 4

Trend: Unknown

Causes ceased?: Unknown

Causes understood?: Unknown

Causes reversible?: Unknown

Extreme fluctuations?: Unknown

#### Area of occupancy

Trend: Unknown

Causes ceased?: Unknown

Causes understood?: Unknown

Causes reversible?: Unknown

Extreme fluctuations?: Unknown

AOO (km2): 4

#### Locations

Number of locations: 1

Justification for number of locations: *Trichoniscoidesouremensis* is recorded from a single cave (Reboleira et al. 2015).

Trend: Stable

Justification for trend: Lapa da Salgada Cave is the only known location for this species; therefore, the trend in number of locations is stable.

Extreme fluctuations?: Unknown

#### Population

Number of individuals: Unknown

Trend: Unknown

Causes ceased?: Unknown

Causes understood?: Unknown

Causes reversible?: Unknown

Extreme fluctuations?: Unknown

#### Subpopulations

Trend: Unknown

Extreme fluctuations?: Unknown

Severe fragmentation?: Unknown

#### Habitat

System: Terrestrial

Habitat specialist: Yes

Habitat (narrative): Lapa da Salgada Cave is composed of a main underground gallery and the floor is mostly covered by flowstone and clay, with a few bat guano deposits.

Trend in extent, area or quality?: Decline (inferred)

##### Habitat

Habitat importance: Major Importance

Habitats: 7.1. Caves and Subterranean Habitats (non-aquatic) - Caves

#### Habitat

Habitat importance: Major Importance

Habitats: 7.1. Caves and Subterranean Habitats (non-aquatic) - Caves

#### Ecology

Size: 3.5 mm (female)

Generation length (yr): 1

Dependency of single sp?: Unknown

Ecology and traits (narrative): *Trichoniscoidesouremensis* is classified as a troglobiont species, being blind and depigmented. It is a single cave endemic (Vandel 1946, Reboleira et al. 2015).

#### Threats

Justification for threats: The cave entrance is located 270 m from a road in which trucks transport goods from warehouses 600 m away. The cave is also located 1 km away from the closest town.

##### Threats

Threat type: Ongoing

Threats: 1.1. Residential & commercial development - Housing & urban areas1.2. Residential & commercial development - Commercial & industrial areas4. Transportation & service corridors9.1. Pollution - Domestic & urban waste water9.2. Pollution - Industrial & military effluents

#### Threats

Threat type: Ongoing

Threats: 1.1. Residential & commercial development - Housing & urban areas1.2. Residential & commercial development - Commercial & industrial areas4. Transportation & service corridors9.1. Pollution - Domestic & urban waste water9.2. Pollution - Industrial & military effluents

#### Conservation

Justification for conservation actions: Measures need to be put in place in order to protect the habitat and species from the disturbances caused by truck movements and proximity to the urban areas.

##### Conservation actions

Conservation action type: Needed

Conservation actions: 1.1. Land/water protection - Site/area protection1.2. Land/water protection - Resource & habitat protection2.1. Land/water management - Site/area management4. Education & awareness5.1.3. Law & policy - Legislation - Sub-national level

#### Conservation actions

Conservation action type: Needed

Conservation actions: 1.1. Land/water protection - Site/area protection1.2. Land/water protection - Resource & habitat protection2.1. Land/water management - Site/area management4. Education & awareness5.1.3. Law & policy - Legislation - Sub-national level

#### Other

##### Use and trade

##### Ecosystem services

#### Use and trade

#### Ecosystem services

#### Viability analysis

### Trichoniscoides serrai

#### Species information

Scientific name: Trichoniscoidesserrai

Species authority: Cruz, 1993

Kingdom: Animalia

Phylum: Arthropoda

Class: Malacostraca

Order: Isopoda

Family: Trichoniscidae

Region for assessment: Europe

#### Editor & Reviewers

##### Reviewers

Reviewers: Reviewers

##### Editor

Editor: Editor

#### Reviewers

Reviewers: Reviewers

#### Editor

Editor: Editor

#### Geographic range

Biogeographic realm: Palearctic

Countries: Portugal

Map of records (Google Earth): Suppl. materials 1, 6

Basis of EOO and AOO: Known habitat extent

Basis (narrative): Both the extent of occurrence (EOO) and area of occupancy (AOO) are 4 km^2^.

Min Elevation/Depth (m): 577

Range description: *Trichoniscoidesserrai* is only recorded from Santo Adrião Cave, located in the palaeokarst of Vimioso in north-eastern Portugal (Cruz 1993, Reboleira et al. 2015).

#### New occurrences

#### Extent of occurrence

EOO (km2): 4

Trend: Unknown

Causes ceased?: Unknown

Causes understood?: Yes

Causes reversible?: Yes

Extreme fluctuations?: Unknown

#### Area of occupancy

Trend: Unknown

Causes ceased?: Unknown

Causes understood?: Unknown

Causes reversible?: Unknown

Extreme fluctuations?: Unknown

AOO (km2): 4

#### Locations

Number of locations: 1

Justification for number of locations: *Trichoniscoidesserrai* occurs in a single cave (Cruz 1993, Reboleira et al. 2015).

Trend: Stable

Justification for trend: Santo Adrião Cave is the only known location for this species; therefore, the trend in number of locations is stable.

Extreme fluctuations?: Unknown

#### Population

Number of individuals: Unknown

Trend: Unknown

Causes ceased?: Unknown

Causes understood?: Unknown

Causes reversible?: Unknown

Extreme fluctuations?: Unknown

#### Subpopulations

Trend: Unknown

Extreme fluctuations?: Unknown

Severe fragmentation?: Unknown

#### Habitat

System: Terrestrial

Habitat specialist: Yes

Habitat (narrative): Santo Adrião mines are located at 580 m a.s.l. in the Municipality of Miranda do Douro, District of Bragança (Moreira and Moreira 2006). The cave consists of a large chamber with visible terraces resulting from the alabaster exploration initiated by the Romans and another smaller chamber that continues through a very narrow passage (Moreira and Moreira 2006).

Trend in extent, area or quality?: Decline (inferred)

##### Habitat

Habitat importance: Major Importance

Habitats: 7.1. Caves and Subterranean Habitats (non-aquatic) - Caves

#### Habitat

Habitat importance: Major Importance

Habitats: 7.1. Caves and Subterranean Habitats (non-aquatic) - Caves

#### Ecology

Size: 3.6 mm (male); 4.2 mm (female)

Generation length (yr): 1

Dependency of single sp?: Unknown

Ecology and traits (narrative): *Trichoniscoidesserrai* is classified as a blind, depigmented troglobiont, adapted to life in the underground (Reboleira et al. 2015).

#### Threats

Justification for threats: The cave entrance is in the border of an active quarry, very close to a road (Moreira and Moreira 2006).

##### Threats

Threat type: Ongoing

Threats: 3.2. Energy production & mining - Mining & quarrying4.1. Transportation & service corridors - Roads & railroads9.2. Pollution - Industrial & military effluents

#### Threats

Threat type: Ongoing

Threats: 3.2. Energy production & mining - Mining & quarrying4.1. Transportation & service corridors - Roads & railroads9.2. Pollution - Industrial & military effluents

#### Conservation

Justification for conservation actions: Even though this cave is protected under legislation by the “Rede Natura 2000” (Directive 1992, ICNB 2000), this species lacks specific protection measures.

##### Conservation actions

Conservation action type: Needed

Conservation actions: 1.1. Land/water protection - Site/area protection1.2. Land/water protection - Resource & habitat protection2.1. Land/water management - Site/area management4. Education & awareness5.1.3. Law & policy - Legislation - Sub-national level

#### Conservation actions

Conservation action type: Needed

Conservation actions: 1.1. Land/water protection - Site/area protection1.2. Land/water protection - Resource & habitat protection2.1. Land/water management - Site/area management4. Education & awareness5.1.3. Law & policy - Legislation - Sub-national level

#### Other

##### Use and trade

##### Ecosystem services

#### Use and trade

#### Ecosystem services

#### Viability analysis

### Trichoniscoides sicoensis

#### Species information

Scientific name: Trichoniscoidessicoensis

Species authority: Reboleira & Taiti 2015

Kingdom: Animalia

Phylum: Arthropoda

Class: Malacostra

Order: Isopoda

Family: Trichoniscidae

Taxonomic notes: *Trichoniscoidessicoensis* is blind and depigmented and it can be distinguished from all species of the genus because the male pereopod 7 merus has a lobe on the mid-sternal margin, the male pleopod 1 exopod has a broadly rounded outer margin and two unequal setae, the endopod has a fusiform distal article with a distinct circular suture in the middle and the male pleopod 2 endopod has a thickset distal article bearing two short triangular lobes and two setae at the apex (Reboleira et al. 2015).

**Figure 1.**
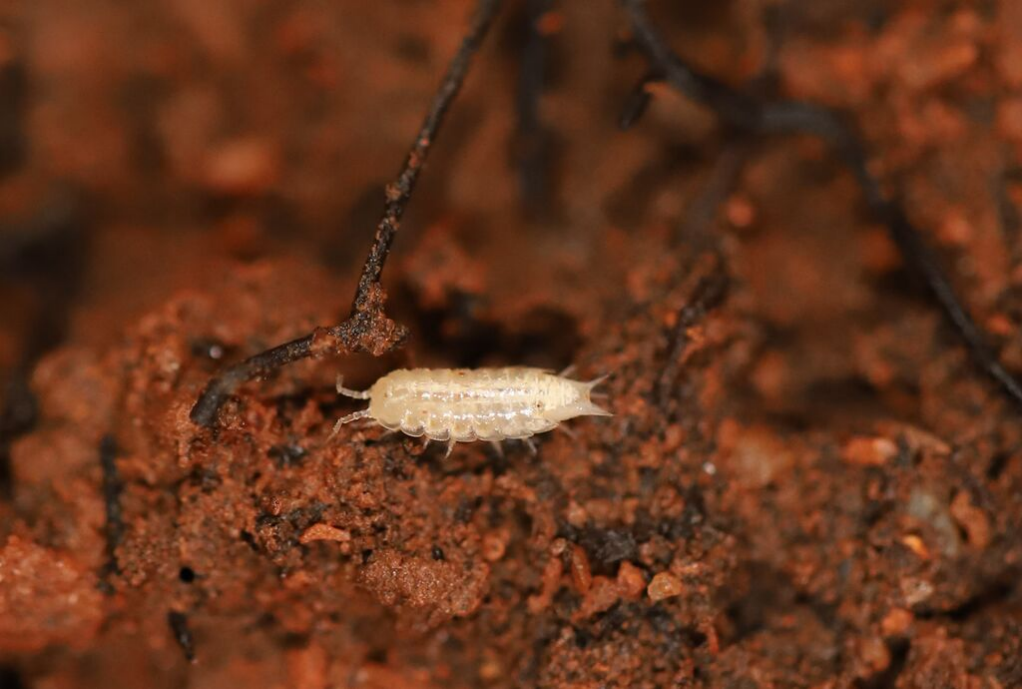
*Trichoniscoidessicoensis* Reboleira & Taiti, 2015 from Cerâmica Cave in Sicó karst area.

Figure(s) or Photo(s): Fig. 1

Region for assessment: Europe

#### Editor & Reviewers

##### Reviewers

Reviewers: Reviewers

##### Editor

Editor: Editor

#### Reviewers

Reviewers: Reviewers

#### Editor

Editor: Editor

#### Geographic range

Biogeographic realm: Palearctic

Countries: Portugal

Map of records (Google Earth): Suppl. materials 1, 7

Basis of EOO and AOO: Known habitat extent

Basis (narrative): The extent of occurrence (EOO) is 129.2 km² and the maximum estimated area of occupancy (AOO) is 20 km².

Min Elevation/Depth (m): 20

Max Elevation/Depth (m): 380

Range description: *Trichoniscoidessicoensis* is recorded from six caves located in the Sicó karst area: Cerâmica, Santa Maria da Estrela, Soprador do Carvalho, Algarinho, Arrifana and São Simão (Reboleira et al. 2015).

#### New occurrences

#### Extent of occurrence

EOO (km2): 129.2

Trend: Unknown

Causes ceased?: Unknown

Causes understood?: Unknown

Causes reversible?: Unknown

Extreme fluctuations?: Unknown

#### Area of occupancy

Trend: Unknown

Causes ceased?: Unknown

Causes understood?: Unknown

Causes reversible?: Unknown

Extreme fluctuations?: Unknown

AOO (km2): 20

#### Locations

Number of locations: 6

Justification for number of locations: *Trichoniscoidessicoensis* is known from six caves (Reboleira et al. 2015).

Trend: Unknown

Extreme fluctuations?: Unknown

#### Population

Number of individuals: Unknown

Trend: Unknown

Causes ceased?: Unknown

Causes understood?: Unknown

Causes reversible?: Unknown

Extreme fluctuations?: Unknown

Population Information (Narrative): The largest population was found in the type locality Cerâmica Cave, where a higher number of specimens has been collected, followed by Santa Maria da Estrela Cave and then Soprador do Carvalho, Algarinho, Arrifana and São Simão Caves (Reboleira et al. 2015).

#### Subpopulations

Number of subpopulations: 6

Trend: Unknown

Extreme fluctuations?: Unknown

Severe fragmentation?: Unknown

#### Habitat

System: Terrestrial

Habitat specialist: Yes

Habitat (narrative): *Trichoniscoidessicoensis* inhabits the deepest and isolated parts of caves of the Sicó karst area, found up to a maximum altitude of 380 m a.s.l. The easternmost locality is Soprador do Carvalho Cave and the westernmost is Santa Maria da Estrela Cave, its distribution being limited at the north by the Arrifana Cave and at the south by the Cerâmica Cave. Cerâmica Cave opens at 355 m a.s.l., it has a horizontal development of 120 m, a depth of 21 m (Nóbrega et al. 1984) and is the richest cave in biodiversity in central Portugal (Reboleira 2012, Reboleira et al. 2015). Santa Maria da Estrela Cave is the habitat recorded at highest altitude, 380 m a.s.l., with a horizontal development of 200 m and harbours frequently a large bat colony (Nóbrega et al. 1984). Soprador do Carvalho Cave is the largest cave, where *T.sicoensis* is distributed and it is part of the Dueça Speleological System, which has 7 km of underground galleries (Neves et al. 2005).

Trend in extent, area or quality?: Decline (inferred)

##### Habitat

Habitat importance: Major Importance

Habitats: 7.1. Caves and Subterranean Habitats (non-aquatic) - Caves

#### Habitat

Habitat importance: Major Importance

Habitats: 7.1. Caves and Subterranean Habitats (non-aquatic) - Caves

#### Ecology

Size: 3.5 mm (male), 3.9 mm (female)

Generation length (yr): 1

Dependency of single sp?: Unknown

Ecology and traits (narrative): *Trichoniscoidessicoensis* is classified as a troglobiont species, blind and depigmented. It is endemic to the Sicó karst area (Reboleira et al. 2015). Most of the specimens are found walking on the substrate near roots, but they can exhibit an amphibious behaviour and have also been collected under a stone submerged in the subterranean stream of Soprador do Carvalho Cave (Reboleira et al. 2015).

#### Threats

Justification for threats: Cerâmica Cave is surrounded by agricultural fields and *Eucalyptus* intensive plantations. It is located 270 m from a road, 550 m from an animal farm, 1.6 km from the closest village and 3.6 km from a quarry. Santa Maria da Estrela Cave is located 86 m from a touristic site called Monstro das Bolachas, 230 m from the Nossa Senhora da Estrela viewpoint, 250 m from the closest urbanised area, 80 m from the road, 220 m from agricultural fields and 2.6 km from two quarries. Soprador do Carvalho Cave is surrounded by agricultural lands distances and is located 67 m from the closest house and 1.4 km from a quarry. This cave is also affected by touristic activities, as people who visit the cave walk all over the habitat (Ribera and Reboleira 2019). The subterranean stream that passes inside this cave has observable urban wastewater run-off as it flows below the urbanised areas of the region (Reboleira et al. 2011). Algarinho Cave entrance is located 170 m from Soprador do Carvalho; therefore, they are both exposed to the same threats. However, the terminus of Algarinho is below a quarry and limestone slurry infiltrates from the surface into the cave, which is known to have pernicious effects on subterranean species (Piccini et al. 2019). Arrifana Cave is located 190 m from a road, 370 m from the nearest village and 900 m from a quarry. São Simão Cave entrance is surrounded by agricultural fields and is located 47 m from a road, 100 m from the closest house and 300 m from a transportation company, from where cargo trucks operate.

##### Threats

Threat type: Ongoing

Threats: 1.1. Residential & commercial development - Housing & urban areas2.2. Agriculture & aquaculture - Wood & pulp plantations3.2. Energy production & mining - Mining & quarrying4. Transportation & service corridors6.1. Human intrusions & disturbance - Recreational activities9.1.2. Pollution - Domestic & urban waste water - Run-off

#### Threats

Threat type: Ongoing

Threats: 1.1. Residential & commercial development - Housing & urban areas2.2. Agriculture & aquaculture - Wood & pulp plantations3.2. Energy production & mining - Mining & quarrying4. Transportation & service corridors6.1. Human intrusions & disturbance - Recreational activities9.1.2. Pollution - Domestic & urban waste water - Run-off

#### Conservation

Justification for conservation actions: Although Cerâmica and Santa Maria da Estrela Caves are protected under legislation by the “Rede Natura 2000” (Directive 1992, ICNB 2000), this troglobiont isopod species is not. It is vital that a conservation plan that takes into consideration both the habitat and the species be developed in order to secure the survival of this species. Of the six caves, only two are protected under legislation. Measures need to be implemented in order to extend this protection to all the caves.The quarry near Algarinho cave has been reported by Grupo Protecção Sicó, a local ONG from Pombal Municipality, as the source of infiltration of large amounts of fine particles of quarry dust in the cave, which puts groundwater quality in danger (Piccini et al. 2019). Prevention of infiltration of wastewaters from villages into the nearby caves is necessary and the effects of the quarries and agriculture in these cave ecosystems need to be minimised.

##### Conservation actions

Conservation action type: Needed

Conservation actions: 1.1. Land/water protection - Site/area protection2.1. Land/water management - Site/area management2.3. Land/water management - Habitat & natural process restoration4. Education & awareness5.1.3. Law & policy - Legislation - Sub-national level

#### Conservation actions

Conservation action type: Needed

Conservation actions: 1.1. Land/water protection - Site/area protection2.1. Land/water management - Site/area management2.3. Land/water management - Habitat & natural process restoration4. Education & awareness5.1.3. Law & policy - Legislation - Sub-national level

#### Other

##### Use and trade

##### Ecosystem services

#### Use and trade

#### Ecosystem services

#### Viability analysis

### Trichoniscoides subterraneus

#### Species information

Scientific name: Trichoniscoidessubterraneus

Species authority: Vandel, 1946

Kingdom: Animalia

Phylum: Arthropoda

Class: Malacostraca

Order: Isopoda

Family: Trichoniscidae

Region for assessment: Europe

#### Editor & Reviewers

##### Reviewers

Reviewers: Reviewers

##### Editor

Editor: Editor

#### Reviewers

Reviewers: Reviewers

#### Editor

Editor: Editor

#### Geographic range

Biogeographic realm: Palearctic

Countries: Portugal

Map of records (Google Earth): Suppl. materials 1, 8

Basis of EOO and AOO: Known habitat extent

Basis (narrative): Both the extent of occurrence (EOO) and area of occupancy (AOO) are 4 km^2^.

Min Elevation/Depth (m): 122

Range description: *Trichoniscoidessubterraneus* is only recorded from Alta do Cabeço dos Mosqueiros Cave, located in Carvalhal de Aljubarrota, the western part of the Estremenho karst massif (Vandel 1946).

#### New occurrences

#### Extent of occurrence

EOO (km2): 4

Trend: Unknown

Causes ceased?: Unknown

Causes understood?: Unknown

Causes reversible?: Unknown

Extreme fluctuations?: Unknown

#### Area of occupancy

Trend: Unknown

Causes ceased?: Unknown

Causes understood?: Unknown

Causes reversible?: Unknown

Extreme fluctuations?: Unknown

AOO (km2): 4

#### Locations

Number of locations: 1

Justification for number of locations: *Trichoniscoidessubterraneus* occurs in a single cave (Reboleira et al. 2015).

Trend: Stable

Justification for trend: Alta do Cabeço dos Mosqueiros Cave is the only known location for this species; therefore, the trend in number of locations is stable.

Extreme fluctuations?: Unknown

#### Population

Number of individuals: Unknown

Trend: Unknown

Causes ceased?: Unknown

Causes understood?: Unknown

Causes reversible?: Unknown

Extreme fluctuations?: Unknown

#### Subpopulations

Trend: Unknown

Extreme fluctuations?: Unknown

Severe fragmentation?: Unknown

#### Habitat

System: Terrestrial

Habitat specialist: Yes

Habitat (narrative): This cave is currently part of the Geocaching network; therefore, it is subject to human disturbance without regulation.

Trend in extent, area or quality?: Decline (inferred)

##### Habitat

Habitat importance: Major Importance

Habitats: 7.1. Caves and Subterranean Habitats (non-aquatic) - Caves

#### Habitat

Habitat importance: Major Importance

Habitats: 7.1. Caves and Subterranean Habitats (non-aquatic) - Caves

#### Ecology

Size: 3 mm

Generation length (yr): 1

Dependency of single sp?: Unknown

Ecology and traits (narrative): *Trichoniscoidessubterraneus* is classified as a blind and depigmented troglobiont species (Reboleira et al. 2015).

#### Threats

Justification for threats: This cave is frequently visited by geocachers looking for the geocache installed inside the cave.

##### Threats

Threat type: Ongoing

Threats: 6.1. Human intrusions & disturbance - Recreational activities

#### Threats

Threat type: Ongoing

Threats: 6.1. Human intrusions & disturbance - Recreational activities

#### Conservation

Justification for conservation actions: Measures to limit the number of visits to the cave should be implemented in order to ensure the integrity of the habitat and the survival of this species.

##### Conservation actions

Conservation action type: Needed

Conservation actions: 1.1. Land/water protection - Site/area protection1.2. Land/water protection - Resource & habitat protection2.1. Land/water management - Site/area management4. Education & awareness5.1.3. Law & policy - Legislation - Sub-national level

#### Conservation actions

Conservation action type: Needed

Conservation actions: 1.1. Land/water protection - Site/area protection1.2. Land/water protection - Resource & habitat protection2.1. Land/water management - Site/area management4. Education & awareness5.1.3. Law & policy - Legislation - Sub-national level

#### Other

##### Use and trade

##### Ecosystem services

#### Use and trade

#### Ecosystem services

#### Viability analysis

### Metatrichoniscoides salirensis

#### Species information

Scientific name: Metatrichoniscoidessalirensis

Species authority: Reboleira & Taiti 2015

Kingdom: Animalia

Phylum: Arthropoda

Class: Malacostraca

Order: Isopoda

Family: Trichoniscidae

Taxonomic notes: *Metatrichoniscoidessalirensis* can be distinguished from all species of the genus because the pleopod 1 exopod of the male has two long distal setae of subequal length and its pleopod 2 endopod has a thickset distal article, ending in a thinner sinuous part with a beak-like small lobe medially directed (Reboleira et al. 2015).

Region for assessment: Europe

#### Editor & Reviewers

##### Reviewers

Reviewers: Reviewers

##### Editor

Editor: Editor

#### Reviewers

Reviewers: Reviewers

#### Editor

Editor: Editor

#### Geographic range

Biogeographic realm: Palearctic

Countries: Portugal

Map of records (Google Earth): Suppl. materials 1, 9

Basis of EOO and AOO: Known habitat extent

Basis (narrative): Both the extent of occurrence (EOO) and area of occupancy (AOO) are 4 km²

Min Elevation/Depth (m): 60

Range description: *Metatrichoniscoidessalirensis* is only known from Salir Cave, a single isolated cave located in the western border of Caldas da Rainha Typhonic Valley (Reboleira et al. 2015).

#### New occurrences

#### Extent of occurrence

EOO (km2): 4

Trend: Unknown

Causes ceased?: Unknown

Causes understood?: Unknown

Causes reversible?: Unknown

Extreme fluctuations?: Unknown

#### Area of occupancy

Trend: Unknown

Causes ceased?: Unknown

Causes understood?: Unknown

Causes reversible?: Unknown

Extreme fluctuations?: Unknown

AOO (km2): 4

#### Locations

Number of locations: 1

Justification for number of locations: *Metatrichoniscoidessalirensis* is only recorded from one cave (Reboleira et al. 2015).

Trend: Stable

Justification for trend: Salir Cave is the only known location for this species; therefore, the trend in number of locations is stable.

Extreme fluctuations?: No

#### Population

Number of individuals: Unknown

Trend: Unknown

Causes ceased?: Unknown

Causes understood?: Unknown

Causes reversible?: Unknown

Extreme fluctuations?: Unknown

Population Information (Narrative): A total of seven specimens have been collected in the type locality (Reboleira et al. 2015).

#### Subpopulations

Trend: Unknown

Extreme fluctuations?: Unknown

Severe fragmentation?: Unknown

#### Habitat

System: Terrestrial

Habitat specialist: Yes

Habitat (narrative): Salir Cave was discovered in the 1960s by the labour force of a quarry. It is located near the sea, at an altitude of 60 m a.s.l. The substrate is mostly composed of flowstone, clay and marine sand that can be found in the deepest parts of the cave (Reboleira et al. 2015).

Trend in extent, area or quality?: Decline (inferred)

##### Habitat

Habitat importance: Major Importance

Habitats: 7.1. Caves and Subterranean Habitats (non-aquatic) - Caves

#### Habitat

Habitat importance: Major Importance

Habitats: 7.1. Caves and Subterranean Habitats (non-aquatic) - Caves

#### Ecology

Size: 2.2 mm (male and female)

Generation length (yr): 1

Dependency of single sp?: Unknown

Ecology and traits (narrative): *Metatrichoniscoidessalirensis* is classified as a troglobiont species, blind and depigmented and is hitherto the only troglobiont species known from this cave and from this karst area (Reboleira et al. 2015). The mean temperature of the cave is 17.3°C, measured in 2010 at the sediment level.

#### Threats

Justification for threats: The cave entrance is located in a former quarry, at 400 m from the closest house and 1 km from the village centre. Salir Cave has an easy access and mostly horizontal development, which attracts casual visitors. It has suffered recurrent episodes of vandalism, which include paintings inside the first chamber and breakage of many lithological formations. This cave is currently listed in the Geocaching network and subject to human disturbance without regulation.

##### Threats

Threat type: Ongoing

Threats: 1.1. Residential & commercial development - Housing & urban areas9.1.2. Pollution - Domestic & urban waste water - Run-off

#### Threats

Threat type: Ongoing

Threats: 1.1. Residential & commercial development - Housing & urban areas9.1.2. Pollution - Domestic & urban waste water - Run-off

#### Conservation

Justification for conservation actions: The cave should be a protected site, as it is the only locality known for this species; therefore, a site of maximum priority for biodiversity conservation. The entrance should be closed and the access regulated in order to prevent the recurrent vandalism and human disturbance to the ecosystem.

##### Conservation actions

Conservation action type: Needed

Conservation actions: 1.1. Land/water protection - Site/area protection1.2. Land/water protection - Resource & habitat protection4. Education & awareness5.1.3. Law & policy - Legislation - Sub-national level

#### Conservation actions

Conservation action type: Needed

Conservation actions: 1.1. Land/water protection - Site/area protection1.2. Land/water protection - Resource & habitat protection4. Education & awareness5.1.3. Law & policy - Legislation - Sub-national level

#### Other

##### Use and trade

##### Ecosystem services

#### Use and trade

#### Ecosystem services

#### Viability analysis

### Troglonethes olissipoensis

#### Species information

Scientific name: Troglonethesolissipoensis

Species authority: Reboleira & Taiti 2015

Kingdom: Animalia

Phylum: Arthropoda

Class: Malacostraca

Order: Isopoda

Family: Trichoniscidae

Taxonomic notes: Antennae have five flagellar articles, the male pleopod 1 exopod is triangular, as wide as long and the male pleopod 2 endopod distal article has a basal and a distal hook-like process (Reboleira et al. 2015).

Region for assessment: Europe

#### Editor & Reviewers

##### Reviewers

Reviewers: Reviewers

##### Editor

Editor: Editor

#### Reviewers

Reviewers: Reviewers

#### Editor

Editor: Editor

#### Geographic range

Biogeographic realm: Palearctic

Countries: Portugal

Map of records (Google Earth): Suppl. materials 1, 10

Basis of EOO and AOO: Known habitat extent

Basis (narrative): Both the extent of occurrence (EOO) and area of occupancy (AOO) are 4 km².

Min Elevation/Depth (m): 42

Range description: *Troglonethesolissipoensis* is only recorded from Alvide Cave, located in Cascais Municipality in the Lisbon metropolitan area (Reboleira et al. 2015).

#### New occurrences

#### Extent of occurrence

EOO (km2): 4

Trend: Unknown

Causes ceased?: Unknown

Causes understood?: Unknown

Causes reversible?: Unknown

Extreme fluctuations?: Unknown

#### Area of occupancy

Trend: Unknown

Causes ceased?: Unknown

Causes understood?: Unknown

Causes reversible?: Unknown

Extreme fluctuations?: Unknown

AOO (km2): 4

#### Locations

Number of locations: 1

Justification for number of locations: *Troglonethesolissipoensis* occurs in a single cave (Reboleira et al. 2015).

Trend: Stable

Justification for trend: Alvide Cave is the only known location for this species; therefore, the trend in number of locations is stable.

Extreme fluctuations?: Unknown

#### Population

Number of individuals: Unknown

Trend: Unknown

Causes ceased?: Unknown

Causes understood?: Unknown

Causes reversible?: Unknown

Extreme fluctuations?: No

Population Information (Narrative): A total of 25 specimens have been collected in the type locality (Reboleira et al. 2015).

#### Subpopulations

Trend: Unknown

Extreme fluctuations?: Unknown

Severe fragmentation?: Unknown

#### Habitat

System: Terrestrial

Habitat specialist: Yes

Habitat (narrative): The entrance to Alvide Cave is through a house currently serving as headquarters of Denível Association. Part of the cave's ceiling has concrete that serves as the base of a residential building. This cave is in the margin of a small canyon and it is composed of three levels of horizontal galleries connected by pits, it has a horizontal development of 708 m and total depth of 28 m.

Trend in extent, area or quality?: Decline (inferred)

##### Habitat

Habitat importance: Major Importance

Habitats: 7.1. Caves and Subterranean Habitats (non-aquatic) - Caves

#### Habitat

Habitat importance: Major Importance

Habitats: 7.1. Caves and Subterranean Habitats (non-aquatic) - Caves

#### Ecology

Size: 3.5 mm (male), 4.2 mm (female)

Generation length (yr): 1

Dependency of single sp?: Unknown

Ecology and traits (narrative): *Troglonethesolissipoensis* is a troglobiont species, being blind and depigmented. It is endemic and the only cave-adapted species known from this cave (Reboleira et al. 2015). The average temperature of the cave is 18.3ºC at soil level and the specimens of *T.olissipoensis* were collected in the deepest parts of the cave (Reboleira et al. 2015).

#### Threats

Justification for threats: Alvide Cave is located below an over-urbanised area (Reboleira et al. 2015).

##### Threats

Threat type: Ongoing

Threats: 1.1. Residential & commercial development - Housing & urban areas9.1.2. Pollution - Domestic & urban waste water - Run-off

#### Threats

Threat type: Ongoing

Threats: 1.1. Residential & commercial development - Housing & urban areas9.1.2. Pollution - Domestic & urban waste water - Run-off

#### Conservation

Justification for conservation actions: Measures should be taken to minimise the pernicious effects due to the cave's close proximity to the urbanisation on the habitat.

##### Conservation actions

Conservation action type: Needed

Conservation actions: 1.1. Land/water protection - Site/area protection1.2. Land/water protection - Resource & habitat protection2.1. Land/water management - Site/area management4. Education & awareness5.1.3. Law & policy - Legislation - Sub-national level

#### Conservation actions

Conservation action type: Needed

Conservation actions: 1.1. Land/water protection - Site/area protection1.2. Land/water protection - Resource & habitat protection2.1. Land/water management - Site/area management4. Education & awareness5.1.3. Law & policy - Legislation - Sub-national level

#### Other

##### Use and trade

##### Ecosystem services

#### Use and trade

#### Ecosystem services

#### Viability analysis

### Troglonethes arrabidaensis

#### Species information

Scientific name: Troglonethesarrabidaensis

Species authority: Reboleira & Taiti 2015

Kingdom: Animalia

Phylum: Arthropoda

Class: Malacostraca

Order: Isopoda

Family: Trichoniscidae

Taxonomic notes: Antennae have three flagellar articles, the male pereopod 7 carpus is enlarged in the basal part, the male pleopod 1 exopod is triangular and as wide as long and the male pleopod 2 endopod has the distal article with an apical hook-like process (Reboleira et al. 2015).

Region for assessment: Europe

#### Editor & Reviewers

##### Reviewers

Reviewers: Reviewers

##### Editor

Editor: Editor

#### Reviewers

Reviewers: Reviewers

#### Editor

Editor: Editor

#### Geographic range

Biogeographic realm: Palearctic

Countries: Portugal

Map of records (Google Earth): Suppl. materials 1, 11

Basis of EOO and AOO: Known habitat extent

Basis (narrative): Both the extent of occurrence (EOO) and area of occupancy (AOO) are 4 km².

Min Elevation/Depth (m): 0

Range description: *Troglonethesarrabidaensis* is only recorded from Frade Cave, located in the Arrábida karst massif (Reboleira et al. 2015).

#### New occurrences

#### Extent of occurrence

EOO (km2): 4

Trend: Unknown

Causes ceased?: Unknown

Causes understood?: Unknown

Causes reversible?: Unknown

Extreme fluctuations?: Unknown

#### Area of occupancy

Trend: Unknown

Causes ceased?: Unknown

Causes understood?: Unknown

Causes reversible?: Unknown

Extreme fluctuations?: Unknown

AOO (km2): 4

#### Locations

Number of locations: 1

Justification for number of locations: *Troglonethesarrabidaensis* occurs in a single cave (Reboleira et al. 2015).

Trend: Stable

Justification for trend: Frade Cave is the only known location for this species; therefore, the trend in number of locations is stable.

Extreme fluctuations?: Unknown

#### Population

Number of individuals: Unknown

Trend: Unknown

Causes ceased?: Unknown

Causes understood?: Unknown

Causes reversible?: Unknown

Extreme fluctuations?: Unknown

Population Information (Narrative): A total of 17 specimens have been collected in Frade Cave (Reboleira et al. 2015).

#### Subpopulations

Trend: Unknown

Extreme fluctuations?: Unknown

Severe fragmentation?: Unknown

#### Habitat

System: Terrestrial

Habitat specialist: Yes

Habitat (narrative): Frade Cave is located near the seashore, with several anchialine lakes inside, which are influenced by the sea tides with a slight delay period (Reboleira et al. 2015). The cave temperature is 21.5°C.

Trend in extent, area or quality?: Decline (inferred)

##### Habitat

Habitat importance: Major Importance

Habitats: 7.1. Caves and Subterranean Habitats (non-aquatic) - Caves

#### Habitat

Habitat importance: Major Importance

Habitats: 7.1. Caves and Subterranean Habitats (non-aquatic) - Caves

#### Ecology

Size: 2.7 mm (male and female)

Generation length (yr): 1

Dependency of single sp?: Unknown

Ecology and traits (narrative): *Troglonethesarrabidaensis* is classified as a troglobiont, depigmented, blind and with an elongated body (Reboleira et al. 2015). It is an endemic and the only cave-adapted species known from this cave so far.

#### Threats

Justification for threats: The cave entrance is located 600 m from a very touristic beach, mainly accessed by boat; therefore, fuel residues brought in by the tidal movement might be a concerning pollutant inside the cave. Contamination of groundwater should be evaluated.

##### Threats

Threat type: Ongoing

Threats: 9. Pollution6.1. Human intrusions & disturbance - Recreational activities

#### Threats

Threat type: Ongoing

Threats: 9. Pollution6.1. Human intrusions & disturbance - Recreational activities

#### Conservation

Justification for conservation actions: Even though this cave is protected under legislation by the “Rede Natura 2000” (Directive 1992, ICNB 2000), this single-cave endemic terrestrial isopod lacks specific protection. Measures should be taken to limit human disturbance in the nearby areas.

##### Conservation actions

Conservation action type: Needed

Conservation actions: 1.1. Land/water protection - Site/area protection1.2. Land/water protection - Resource & habitat protection2.1. Land/water management - Site/area management4. Education & awareness5.1.3. Law & policy - Legislation - Sub-national level

#### Conservation actions

Conservation action type: Needed

Conservation actions: 1.1. Land/water protection - Site/area protection1.2. Land/water protection - Resource & habitat protection2.1. Land/water management - Site/area management4. Education & awareness5.1.3. Law & policy - Legislation - Sub-national level

#### Other

##### Use and trade

##### Ecosystem services

#### Use and trade

#### Ecosystem services

#### Viability analysis

### Miktoniscus longispina

#### Species information

Scientific name: Miktoniscuslongispina

Species authority: Reboleira & Taiti 2015

Kingdom: Animalia

Phylum: Arthropoda

Class: Malacostraca

Order: Isopoda

Family: Trichoniscidae

Taxonomic notes: The male pereopod 7 has a long and stout seta on the distal corner of the ischium and a triangular male pleopod 1 exopod (Reboleira et al. 2015). The specimens from the Sicó karst caves lack eyes, while those from Bolhos have a single black ocellus (Reboleira et al. 2015).

**Figure 2.**
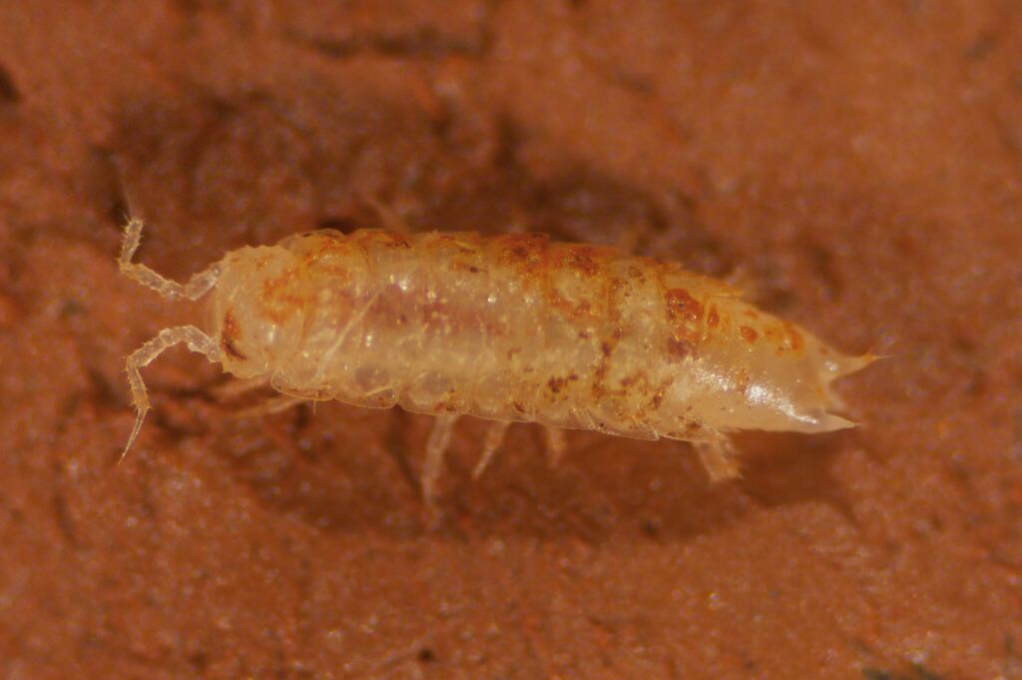
*Miktoniscuslongispina* Reboleira & Taiti, 2015 from Cerâmica Cave in Sicó karst area.

Figure(s) or Photo(s): Fig. 2

Region for assessment: Europe

#### Editor & Reviewers

##### Reviewers

Reviewers: Reviewers

##### Editor

Editor: Editor

#### Reviewers

Reviewers: Reviewers

#### Editor

Editor: Editor

#### Geographic range

Biogeographic realm: Palearctic

Countries: Portugal

Map of records (Google Earth): Suppl. materials 1, 12

Basis of EOO and AOO: Known habitat extent

Basis (narrative): The extent of occurrence (EOO) is 137.96 km² and the area of occupancy (AOO) is 12 km².

Min Elevation/Depth (m): 145

Max Elevation/Depth (m): 355

Range description: *Miktoniscuslongispina* is recorded from three caves with disjunt distribution. Bolhos Cave, also known as Casal da Lebre Cave, located in the Cesaredas Plateau and Ervilha and Cerâmica Caves located in the centre of the Sicó karst area (Reboleira et al. 2015).

#### New occurrences

#### Extent of occurrence

EOO (km2): 137.96

Trend: Unknown

Causes ceased?: Unknown

Causes understood?: Unknown

Causes reversible?: Unknown

Extreme fluctuations?: Unknown

#### Area of occupancy

Trend: Unknown

Causes ceased?: Unknown

Causes understood?: Unknown

Causes reversible?: Unknown

Extreme fluctuations?: Unknown

AOO (km2): 12

#### Locations

Number of locations: 3

Justification for number of locations: *Miktoniscuslongispina* can be found in three caves (Reboleira et al. 2015).

Trend: Unknown

Extreme fluctuations?: Unknown

#### Population

Number of individuals: Unknown

Trend: Unknown

Causes ceased?: Unknown

Causes understood?: Unknown

Causes reversible?: Unknown

Extreme fluctuations?: Unknown

Population Information (Narrative): A total of 12 specimens were collected in the three localities: two in Bolhos Cave, eight in Cerâmica Cave and two in Algar da Ervilha Cave (Reboleira et al. 2015).

#### Subpopulations

Number of subpopulations: 3

Trend: Unknown

Extreme fluctuations?: Unknown

Severe fragmentation?: Yes

#### Habitat

System: Terrestrial

Habitat specialist: Yes

Habitat (narrative): Bolhos Cave has a horizontal development of 130 m and is located in Cesaredas Plateau, 93 km away from the other localities in the Sicó karst area. Cerâmica Cave has a horizontal development of 120 m and is the richest cave in troglobiont species in central Portugal. Algar da Ervilha Cave has a depth of 52 m and a horizontal development of 150 m (Nóbrega et al. 1984). The cave temperatures range between 15°C (in Cerâmica) and 16.3°C (in Bolhos).

Trend in extent, area or quality?: Decline (inferred)

##### Habitat

Habitat importance: Major Importance

Habitats: 7.1. Caves and Subterranean Habitats (non-aquatic) - Caves

#### Habitat

Habitat importance: Major Importance

Habitats: 7.1. Caves and Subterranean Habitats (non-aquatic) - Caves

#### Ecology

Size: 3.5 mm (male and female)

Generation length (yr): 1

Dependency of single sp?: Unknown

Ecology and traits (narrative): *Miktoniscuslongispina* is a troglobiont, with a depigmented and elongated body. All specimens, except for the ones collected in Bolhos Cave, are blind.

#### Threats

Justification for threats: Bolhos Cave is located 130 m from an energy windmill and 700 m from the closest village, in an area of extensive agricultural fields. Cerâmica Cave is surrounded by agricultural fields and *Eucalyptus* intensive plantations. It is located 270 m from a road, 550 m from an animal farm, 1.6 km from the closest village and 3.6 km from a quarry. Algar da Ervilha Cave is located in the border of a road in the westernmost access to Ereiras Village, at 90 m from the closest house and 3.5 km from the same quarry as Cerâmica Cave.

##### Threats

Threat type: Ongoing

Threats: 1.1. Residential & commercial development - Housing & urban areas2.2. Agriculture & aquaculture - Wood & pulp plantations2.3. Agriculture & aquaculture - Livestock farming & ranching3.2. Energy production & mining - Mining & quarrying3.3. Energy production & mining - Renewable energy4. Transportation & service corridors9.1.2. Pollution - Domestic & urban waste water - Run-off

#### Threats

Threat type: Ongoing

Threats: 1.1. Residential & commercial development - Housing & urban areas2.2. Agriculture & aquaculture - Wood & pulp plantations2.3. Agriculture & aquaculture - Livestock farming & ranching3.2. Energy production & mining - Mining & quarrying3.3. Energy production & mining - Renewable energy4. Transportation & service corridors9.1.2. Pollution - Domestic & urban waste water - Run-off

#### Conservation

Justification for conservation actions: The caves located in the Sicó karst area are protected under the “Rede Natura 2000” (Directive 1992, ICNB 2000). Bolhos Cave is not protected under legislation, urging adequate protection. Measures should be taken to prevent infiltration from agricultural lands, livestock farms and villages and to prevent the pernicious effects resulting from the nearby quarries and windmills.

##### Conservation actions

Conservation action type: Needed

Conservation actions: 1.1. Land/water protection - Site/area protection1.2. Land/water protection - Resource & habitat protection2.1. Land/water management - Site/area management4. Education & awareness5.1.3. Law & policy - Legislation - Sub-national level

#### Conservation actions

Conservation action type: Needed

Conservation actions: 1.1. Land/water protection - Site/area protection1.2. Land/water protection - Resource & habitat protection2.1. Land/water management - Site/area management4. Education & awareness5.1.3. Law & policy - Legislation - Sub-national level

#### Other

##### Use and trade

##### Ecosystem services

#### Use and trade

#### Ecosystem services

#### Viability analysis

### Moserius inexpectatus

#### Species information

Scientific name: Moseriusinexpectatus

Species authority: Reboleira & Taiti 2015

Kingdom: Animalia

Phylum: Arthropoda

Class: Malacostraca

Order: Isopoda

Family: Trichoniscidae

Taxonomic notes: The male pereopod 7 carpus has a distal lobe on the sternal margin. This new species is easily distinguishable from the other two *Moserius* species due to the peculiar shape of the male pleopod 1 exopod, with a truncate and sinuous, rather than triangular, distal point (Reboleira et al. 2015).

Region for assessment: Europe

#### Editor & Reviewers

##### Reviewers

Reviewers: Reviewers

##### Editor

Editor: Editor

#### Reviewers

Reviewers: Reviewers

#### Editor

Editor: Editor

#### Geographic range

Biogeographic realm: Palearctic

Countries: Portugal

Map of records (Google Earth): Suppl. materials 1, 13

Basis of EOO and AOO: Known habitat extent

Basis (narrative): Both the extent of occurrence (EOO) and area of occupancy (AOO) are 4 km^2^.

Min Elevation/Depth (m): 95

Range description: *Moseriusinexpectatus* is only recorded from Almonda Cave, also known as Olho do Moinho da Fonte, located in the easternmost subunit of the Estremenho karst massif (Reboleira et al. 2015).

#### New occurrences

#### Extent of occurrence

EOO (km2): 4

Trend: Unknown

Causes ceased?: Unknown

Causes understood?: Unknown

Causes reversible?: Unknown

Extreme fluctuations?: Unknown

#### Area of occupancy

Trend: Unknown

Causes ceased?: Unknown

Causes understood?: Unknown

Causes reversible?: Unknown

Extreme fluctuations?: Unknown

AOO (km2): 4

#### Locations

Number of locations: 1

Justification for number of locations: *Moseriusinexpectatus* is endemic to a single cave (Reboleira et al. 2015).

Trend: Stable

Justification for trend: Almonda Cave is the only location from where this species is known; therefore, the trend in number of locations is stable.

Extreme fluctuations?: Unknown

#### Population

Number of individuals: Unknown

Trend: Unknown

Causes ceased?: Unknown

Causes understood?: Unknown

Causes reversible?: Unknown

Extreme fluctuations?: Unknown

Population Information (Narrative): Only one specimen was collected from the type locality (Reboleira et al. 2015).

#### Subpopulations

Trend: Unknown

Extreme fluctuations?: Unknown

Severe fragmentation?: Unknown

#### Habitat

System: Terrestrial

Habitat specialist: Yes

Habitat (narrative): Almonda Cave, the type locality for this species, is the largest cave in Portugal (Thomas 1991).

Trend in extent, area or quality?: Decline (inferred)

##### Habitat

Habitat importance: Major Importance

Habitats: 7.1. Caves and Subterranean Habitats (non-aquatic) - Caves

#### Habitat

Habitat importance: Major Importance

Habitats: 7.1. Caves and Subterranean Habitats (non-aquatic) - Caves

#### Ecology

Size: 1.5 mm (male)

Generation length (yr): 1

Dependency of single sp?: Unknown

Ecology and traits (narrative): *Moseriusinexpectatus* is a troglobiont blind and depigmented isopod. This is the third species described from the genus *Moserius*, previously known from Slovenia and Italy (Reboleira et al. 2015).

#### Threats

Justification for threats: Almonda Cave is located 50 m from a factory that extracts and uses water from a subterranean stream and 420 m from a village, which has many agricultural fields.

##### Threats

Threat type: Ongoing

Threats: 1.1. Residential & commercial development - Housing & urban areas1.2. Residential & commercial development - Commercial & industrial areas2.1. Agriculture & aquaculture - Annual & perennial non-timber crops9.1.2. Pollution - Domestic & urban waste water - Run-off

#### Threats

Threat type: Ongoing

Threats: 1.1. Residential & commercial development - Housing & urban areas1.2. Residential & commercial development - Commercial & industrial areas2.1. Agriculture & aquaculture - Annual & perennial non-timber crops9.1.2. Pollution - Domestic & urban waste water - Run-off

#### Conservation

Justification for conservation actions: In 1993, Almonda Cave was classified as a Property of Public Interest (IIP) and protected due to its archaeological heritage (Hoffmann et al. 2013). The archaeological arguments are, however, inappropriate for cave-adapted fauna protection, so it is urgent to set protective measures directed at the cave fauna. Measures also need to be established to protect the habitats and locations that are affected by human activities, wastewater infiltration and pollution.

##### Conservation actions

Conservation action type: Needed

Conservation actions: 1.1. Land/water protection - Site/area protection1.2. Land/water protection - Resource & habitat protection2.1. Land/water management - Site/area management4. Education & awareness5.1.3. Law & policy - Legislation - Sub-national level

#### Conservation actions

Conservation action type: Needed

Conservation actions: 1.1. Land/water protection - Site/area protection1.2. Land/water protection - Resource & habitat protection2.1. Land/water management - Site/area management4. Education & awareness5.1.3. Law & policy - Legislation - Sub-national level

#### Other

##### Use and trade

##### Ecosystem services

#### Use and trade

#### Ecosystem services

#### Viability analysis

### Cordioniscus lusitanicus

#### Species information

Scientific name: Cordioniscuslusitanicus

Species authority: Reboleira & Taiti 2015

Kingdom: Animalia

Phylum: Arthropoda

Class: Malacostraca

Order: Isopoda

Family: Styloniscidae

Taxonomic notes: The male pereopod 7 ischium has a rounded hyaline basal lobe, the triangular male pleopod 1 exopod is as long as the endopod and the male pleopod 2 endopod has a complex apical part (Reboleira et al. 2015).

Region for assessment: Europe

#### Editor & Reviewers

##### Reviewers

Reviewers: Reviewers

##### Editor

Editor: Editor

#### Reviewers

Reviewers: Reviewers

#### Editor

Editor: Editor

#### Geographic range

Biogeographic realm: Palearctic

Countries: Portugal

Map of records (Google Earth): Suppl. materials 1, 14

Basis of EOO and AOO: Known habitat extent

Basis (narrative): The extent of occurrence (EOO) is 5,893.93 km^2^ and the area of occupancy (AOO) is 16 km^2^.

Min Elevation/Depth (m): 10

Max Elevation/Depth (m): 370

Range description: *Cordioniscuslusitanicus* is recorded from five caves, located in two isolated karst areas: Algar de Santo António from the Estremoz-Cano karst massif and Ibne Ammar, Algarão do Remexido, Vale Telheiro and Senhora Caves from the Algarve karst massif (Reboleira et al. 2015).

#### New occurrences

#### Extent of occurrence

EOO (km2): 5,893.93

Trend: Unknown

Justification for trend: The four caves, located in the Algarve karst massif, are at 200 km distance from the cave in the Estremoz-Cano karst massif.

Causes ceased?: Unknown

Causes understood?: Unknown

Causes reversible?: Unknown

Extreme fluctuations?: Unknown

#### Area of occupancy

Trend: Unknown

Causes ceased?: Unknown

Causes understood?: Unknown

Causes reversible?: Unknown

Extreme fluctuations?: Unknown

AOO (km2): 16

#### Locations

Number of locations: 5

Justification for number of locations: *Cordioniscuslusitanicus* occurs in five caves (Reboleira et al. 2015).

Trend: Unknown

Extreme fluctuations?: Unknown

#### Population

Number of individuals: Unknown

Trend: Unknown

Causes ceased?: Unknown

Causes understood?: Unknown

Causes reversible?: Unknown

Extreme fluctuations?: Unknown

Population Information (Narrative): A total of 26 specimens have been collected: nine from Algar de Santo António, six from Ibne Ammar, six from Algarão do Remexido and four from Senhora (Reboleira et al. 2015) and one from Vale Telheiro.

#### Subpopulations

Number of subpopulations: 5

Trend: Unknown

Extreme fluctuations?: Unknown

Severe fragmentation?: Unknown

#### Habitat

System: Terrestrial

Habitat specialist: Unknown

Habitat (narrative): *Cordioniscuslusitanicus* was collected in two karst areas, Estremoz-Cano and Algarve, located more than 200 km apart, which are isolated from each other by the dry and flat areas of the Alentejo Province. In Algar de Santo António, the specimens were collected in deep layers of soil at the bottom of an entrance pit of the cave (Reboleira et al. 2015).

Trend in extent, area or quality?: Decline (inferred)

##### Habitat

Habitat importance: Major Importance

Habitats: 7.1. Caves and Subterranean Habitats (non-aquatic) - Caves

#### Habitat

Habitat importance: Major Importance

Habitats: 7.1. Caves and Subterranean Habitats (non-aquatic) - Caves

#### Ecology

Size: 3 mm (female), 5 mm (male)

Generation length (yr): 1

Dependency of single sp?: Unknown

Ecology and traits (narrative): *Cordioniscuslusitanicus* is classified as a troglobiont and endogean species. It is blind, depigmented and has an elongated body (Reboleira et al. 2015).

#### Threats

Justification for threats: Algar de Santo António is located in the middle of an urbanised area of a village. Algarão do Remexido is located under agricultural lands, 370 m from the closest house and 1.7 km from the closest village. Senhora Cave is located 168 m from the closest house and 900 m from an industrial complex. Ibne Ammar Cave is located right in the flooding zone of the Arade river, 380 m from the national road IC4 and 1.4 km from the nearest town. Vale Telheiro Cave is located 50 m from a road and 150 m from the closest house.

##### Threats

Threat type: Ongoing

Threats: 1.1. Residential & commercial development - Housing & urban areas1.2. Residential & commercial development - Commercial & industrial areas2.2. Agriculture & aquaculture - Wood & pulp plantations4. Transportation & service corridors9.1.2. Pollution - Domestic & urban waste water - Run-off

#### Threats

Threat type: Ongoing

Threats: 1.1. Residential & commercial development - Housing & urban areas1.2. Residential & commercial development - Commercial & industrial areas2.2. Agriculture & aquaculture - Wood & pulp plantations4. Transportation & service corridors9.1.2. Pollution - Domestic & urban waste water - Run-off

#### Conservation

Justification for conservation actions: Of the five caves, only Ibne Ammar Cave is protected under legislation by the “Rede Natura 2000” (Directive 1992, ICNB 2000) and this troglobiont isopod species is not considered for the protection measures.Population trends need to be monitored in order to better understand the species abundance patterns and life cycle and the species evolution in two isolated massifs. Measures to prevent infiltration of wastewaters and agricultural and industrial contamination need to be taken to ensure the proper conservation of the natural landscape, vital to the nutrient flow to the subterranean ecosystems.

##### Conservation actions

Conservation action type: Needed

Conservation actions: 1.1. Land/water protection - Site/area protection1.2. Land/water protection - Resource & habitat protection2.1. Land/water management - Site/area management4. Education & awareness5.1.3. Law & policy - Legislation - Sub-national level

#### Conservation actions

Conservation action type: Needed

Conservation actions: 1.1. Land/water protection - Site/area protection1.2. Land/water protection - Resource & habitat protection2.1. Land/water management - Site/area management4. Education & awareness5.1.3. Law & policy - Legislation - Sub-national level

#### Other

##### Use and trade

##### Ecosystem services

#### Use and trade

#### Ecosystem services

#### Viability analysis

### Porcellio cavernicolus

#### Species information

Scientific name: Porcelliocavernicolus

Species authority: Vandel, 1946

Kingdom: Animalia

Phylum: Arthropoda

Class: Malacostraca

Order: Isopoda

Family: Porcellionidae

**Figure 3.**
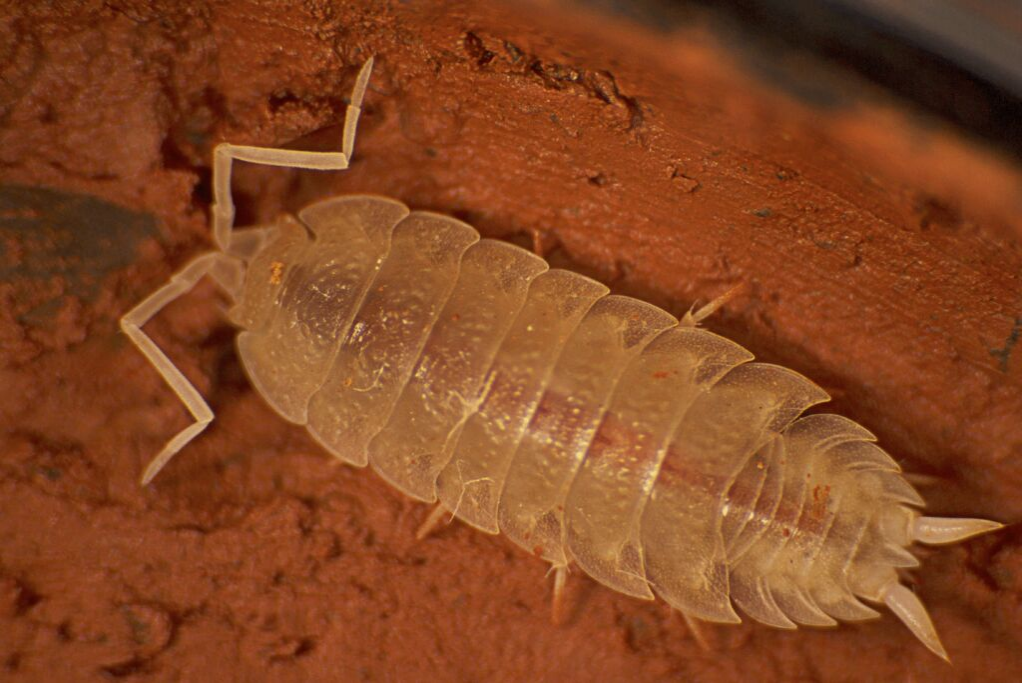
*Porcelliocavernicolus* Vandel, 1946 from Arrifana Cave in Sicó karst area.

Figure(s) or Photo(s): Fig. 3

Region for assessment: Europe

#### Editor & Reviewers

##### Reviewers

Reviewers: Reviewers

##### Editor

Editor: Editor

#### Reviewers

Reviewers: Reviewers

#### Editor

Editor: Editor

#### Geographic range

Biogeographic realm: Palearctic

Countries: Portugal

Map of records (Google Earth): Suppl. materials 1, 15

Basis of EOO and AOO: Known habitat extent

Basis (narrative): The extent of occurrence (EOO) is 713 km^2^ and the area of occupancy (AOO) is 24 km^2^.

Min Elevation/Depth (m): 20

Max Elevation/Depth (m): 380

Range description: *Porcelliocavernicolus* is recorded from seven caves located in two isolated massifs, Gruta d’el Rey in the Cantanhede-Outil karst massif and Santa Maria da Estrela, Soprador do Carvalho, Algarinho, Cerâmica, Abrigo Tomar I and Furjaca caves, located in the Sicó massif (Reboleira et al. 2015).

#### New occurrences

#### Extent of occurrence

EOO (km2): 713

Trend: Unknown

Causes ceased?: Unknown

Causes understood?: Unknown

Causes reversible?: Unknown

Extreme fluctuations?: Unknown

#### Area of occupancy

Trend: Unknown

Causes ceased?: Unknown

Causes understood?: Unknown

Causes reversible?: Unknown

Extreme fluctuations?: Unknown

AOO (km2): 24

#### Locations

Number of locations: 7

Justification for number of locations: *Porcelliocavernicolus* occurs in seven caves (Reboleira et al. 2015).

Trend: Unknown

Extreme fluctuations?: Unknown

#### Population

Number of individuals: Unknown

Trend: Unknown

Causes ceased?: Unknown

Causes understood?: Unknown

Causes reversible?: Unknown

Extreme fluctuations?: Unknown

Population Information (Narrative): More than 63 specimens have been collected: 28 from Gruta d’el Rey, 16 from Santa Maria da Estrela, seven from Soprador do Carvalho, three from Cerâmica, four from Abrigo Tomar I and five from Furjaca. The specimens, collected from Algarinho Cave, are simply described as “many” (Reboleira et al. 2015).

#### Subpopulations

Number of subpopulations: 7

Trend: Unknown

Extreme fluctuations?: Unknown

Severe fragmentation?: Unknown

#### Habitat

System: Terrestrial

Habitat specialist: Yes

Habitat (narrative): *Porcelliocavernicolus* inhabits the most superficial parts of caves and it occurs on roots that hang from the ceiling. Specimens are easily distinguishable due to their whitish colouration (Reboleira et al. 2015).

Trend in extent, area or quality?: Decline (inferred)

##### Habitat

Habitat importance: Major Importance

Habitats: 7.1. Caves and Subterranean Habitats (non-aquatic) - Caves

#### Habitat

Habitat importance: Major Importance

Habitats: 7.1. Caves and Subterranean Habitats (non-aquatic) - Caves

#### Ecology

Size: 10 mm (male and female)

Generation length (yr): 1

Dependency of single sp?: Unknown

Ecology and traits (narrative): *Porcelliocavernicolus* is classifid as a troglobiont, endemic to seven caves from central Portugal, distributed in two isolated karst massifs (Reboleira et al. 2015).

#### Threats

Justification for threats: Gruta d‘el Rey is located in the middle of an urbanised area, 1 km from a quarry and 1.2 km from highway A14. Santa Maria da Estrela Cave is located 86 m from a touristic site called Monstro das Bolachas, 230 m from the Nossa Senhora da Estrela viewpoint, 250 m from the closest house, 80 m from the closest road, 220 m from agricultural fields and 2.6 km from two quarries. Soprador do Carvalho is surrounded by agricultural lands and is located 67 m from the closest house and 1.4 km from a quarry. This cave is also affected by touristic activities, as people who visit the cave walk all over the habitat (Ribera and Reboleira 2019). The subterranean stream that passes inside this cave has observable urban wastewater run-off as it flows below the urbanised areas of the region (Reboleira et al. 2011). Algarinho Cave entrance is located 170 m from the entrance of Soprador do Carvalho Cave; therefore, they are both exposed to similar threats. Cerâmica Cave is surrounded by agricultural fields and *Eucalyptus* intensive plantations. It is located 270 m from a road, 550 m from an animal farm, 1.6 km from the closest village and 3.6 km from a quarry. Abrigo Tomar I Cave is located in a quite pristine location and it is a local protected area by the ONG Quercus. Furjaca Cave is located in an abandoned quarry which, after closure, was used as a trash dumping site.

##### Threats

Threat type: Ongoing

Threats: 1.1. Residential & commercial development - Housing & urban areas1.2. Residential & commercial development - Commercial & industrial areas2.2. Agriculture & aquaculture - Wood & pulp plantations3.2. Energy production & mining - Mining & quarrying4. Transportation & service corridors6.1. Human intrusions & disturbance - Recreational activities9.1. Pollution - Domestic & urban waste water9.4. Pollution - Garbage & solid waste

#### Threats

Threat type: Ongoing

Threats: 1.1. Residential & commercial development - Housing & urban areas1.2. Residential & commercial development - Commercial & industrial areas2.2. Agriculture & aquaculture - Wood & pulp plantations3.2. Energy production & mining - Mining & quarrying4. Transportation & service corridors6.1. Human intrusions & disturbance - Recreational activities9.1. Pollution - Domestic & urban waste water9.4. Pollution - Garbage & solid waste

#### Conservation

Justification for conservation actions: Of the seven locations, only three are protected under legislation by the “Rede Natura 2000” (Directive 1992, ICNB 2000). Almost all of the locations are severely disturbed by human activities; therefore, preventative measures and conservation actions need to be established in order to stop the devastating effects of these anthropogenic threats to the habitat and species.

##### Conservation actions

Conservation action type: Needed

Conservation actions: 1.1. Land/water protection - Site/area protection1.2. Land/water protection - Resource & habitat protection2.1. Land/water management - Site/area management4. Education & awareness5.1.3. Law & policy - Legislation - Sub-national level

#### Conservation actions

Conservation action type: Needed

Conservation actions: 1.1. Land/water protection - Site/area protection1.2. Land/water protection - Resource & habitat protection2.1. Land/water management - Site/area management4. Education & awareness5.1.3. Law & policy - Legislation - Sub-national level

#### Other

##### Use and trade

##### Ecosystem services

#### Use and trade

#### Ecosystem services

#### Viability analysis

### Trogleluma machadoi

#### Species information

Scientific name: Troglelumamachadoi

Species authority: (Vandel, 1946)

Kingdom: Animalia

Phylum: Arthropoda

Class: Malacostraca

Order: Isopoda

Family: Armadillidiidae

**Figure 4.**
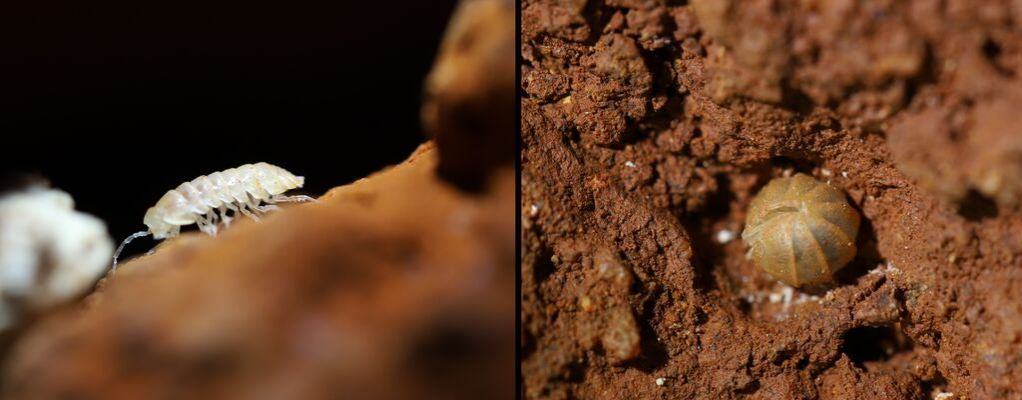
*Troglelumamachadoi* (Vandel, 1946) from Senhora Cave in Cerro da Cabeça, Moncarapacho, Algarve.

Figure(s) or Photo(s): Fig. 4

Region for assessment: Europe

#### Editor & Reviewers

##### Reviewers

Reviewers: Reviewers

##### Editor

Editor: Editor

#### Reviewers

Reviewers: Reviewers

#### Editor

Editor: Editor

#### Geographic range

Biogeographic realm: Palearctic

Countries: Portugal

Map of records (Google Earth): Suppl. materials 1, 16

Basis of EOO and AOO: Known habitat extent

Basis (narrative): The extent of occurrence (EOO) is 356.4 km^2^ and the area of occupancy (AOO) is 16 km^2^.

Min Elevation/Depth (m): 10

Max Elevation/Depth (m): 260

Range description: *Troglelumamachadoi* is recorded from six caves, located in the Algarve karst massif: Ibne Ammar, Algarão do Remexido, Vale Telheiro, Senhora, Algarão do Paulino and Abismo Novo (Reboleira et al. 2015).

#### New occurrences

#### Extent of occurrence

EOO (km2): 356.4

Trend: Unknown

Causes ceased?: Unknown

Causes understood?: Unknown

Causes reversible?: Unknown

Extreme fluctuations?: Unknown

#### Area of occupancy

Trend: Unknown

Causes ceased?: Unknown

Causes understood?: Unknown

Causes reversible?: Unknown

Extreme fluctuations?: Unknown

AOO (km2): 16

#### Locations

Number of locations: 6

Justification for number of locations: *Troglelumamachadoi* occurs in six caves (Reboleira et al. 2015).

Trend: Unknown

Extreme fluctuations?: Unknown

#### Population

Number of individuals: Unknown

Trend: Unknown

Causes ceased?: Unknown

Causes understood?: Unknown

Causes reversible?: Unknown

Extreme fluctuations?: Unknown

#### Subpopulations

Number of subpopulations: 6

Trend: Unknown

Extreme fluctuations?: Unknown

Severe fragmentation?: Unknown

#### Habitat

System: Terrestrial

Habitat specialist: Yes

Habitat (narrative): *Troglelumamachadoi* is endemic to karst caves in the southernmost province of Portugal, the Algarve.

Trend in extent, area or quality?: Decline (inferred)

##### Habitat

Habitat importance: Major Importance

Habitats: 7.1. Caves and Subterranean Habitats (non-aquatic) - Caves

#### Habitat

Habitat importance: Major Importance

Habitats: 7.1. Caves and Subterranean Habitats (non-aquatic) - Caves

#### Ecology

Size: 8 mm

Generation length (yr): 1

Dependency of single sp?: Unknown

Ecology and traits (narrative): *Troglelumamachadoi* is a blind and depigmented species, classified as a troglobiont. Specimens are found in the most deep and well isolated parts of of caves, they have the integument covered with clay, while some others were found walking on cave walls and completely clean of clay, probably recently moulted (Reboleira et al. 2015, Vandel 1946). The temperature of the caves in the distribution localities ranges from 17 to 20.5°C, measured at the sediment level. The species is distributed from 10 m a.s.l., at Ibne Ammar Cave, up to a maximum altitude recorded of 239 m a.s.l. *Troglelumamachadoi* shares habitat with some iconic cave-adapted species from the Algarve, such as: the giant pseudoscorpion *Titanobochicamagna* (Reboleira et al. 2010b), the relictual pseudoscorpion *Lusoblothrusaenigmaticus* (Reboleira et al. 2012b), the dysderid spider *Harpacteastalitoides* (Ribera 1993), the millipedes *Acipesbifilum* and *A.machadoi* and *Boreviulisomabarrocalense* (Enghoff and Reboleira 2013, Reboleira and Enghoff 2013), the dipluran *Litocampamendesi* (Reboleira et al. 2019), the giant silverfish *Squamatiniaalgharbica* (Reboleira et al. 2012a) and the beetle *Speonemadusalgarvensis* (Reboleira et al. 2017).

#### Threats

Justification for threats: Ibne Ammar Cave is located right in the flooding zone of the Arade River, 380 m from the national road IC4 and 1.4 km from the nearest town. Algarão do Remexido is located under agricultural lands, 370 m from the closest house and 1.7 km from the closest village. Senhora Cave is located 168 m from the closest house and 900 m from an industrial complex. Abismo Novo Cave is located 100 m from the closest house and 500 m from the village centre and is also located 1 km from Senhora Cave. Algarão do Paulino is located near a road, 90 m from the closest house and 800 m from the closest village. Vale Telheiro Cave is located 50 m from a road and 150 m from the closest house.

##### Threats

Threat type: Ongoing

Threats: 1.1. Residential & commercial development - Housing & urban areas1.2. Residential & commercial development - Commercial & industrial areas2.1. Agriculture & aquaculture - Annual & perennial non-timber crops4. Transportation & service corridors9.1.2. Pollution - Domestic & urban waste water - Run-off

#### Threats

Threat type: Ongoing

Threats: 1.1. Residential & commercial development - Housing & urban areas1.2. Residential & commercial development - Commercial & industrial areas2.1. Agriculture & aquaculture - Annual & perennial non-timber crops4. Transportation & service corridors9.1.2. Pollution - Domestic & urban waste water - Run-off

#### Conservation

Justification for conservation actions: Of the five locations, only three are protected under legislation by the “Rede Natura 2000” (Directive 1992, ICNB 2000). Measures should be taken to prevent infiltration of wastewaters from villages into the nearby caves and to minimise the effects of anthropogenic threats on the habitats and species.

##### Conservation actions

Conservation action type: Needed

Conservation actions: 1.1. Land/water protection - Site/area protection1.2. Land/water protection - Resource & habitat protection2.1. Land/water management - Site/area management4. Education & awareness5.1.3. Law & policy - Legislation - Sub-national level

#### Conservation actions

Conservation action type: Needed

Conservation actions: 1.1. Land/water protection - Site/area protection1.2. Land/water protection - Resource & habitat protection2.1. Land/water management - Site/area management4. Education & awareness5.1.3. Law & policy - Legislation - Sub-national level

#### Other

##### Use and trade

##### Ecosystem services

#### Use and trade

#### Ecosystem services

#### Viability analysis

## Discussion

Cave-adapted terrestrial isopods are key species for cave ecosystem conservation: i) they are the most diverse group of cave-adapted species in continental Portugal, ii) they have several single cave endemics that are under threat and require specific protection measures, iii) they are basal in the trophic chains in caves and serve as a food source for several other zoological groups; iv) they play a vital role on the decomposition of organic matter in caves; and v) they are very sensitive to contaminants and climate change (Campos-Filho et al. 2014, van Gestel et al. 2018, Reboleira et al. 2015).

Almost all cave-adapted terrestrial isopod species face direct anthropogenic threats, such as point or diffuse pollution, direct habitat destruction by mining and quarry activities or excess cave visitation (Reboleira et al. 2011, Reboleira 2012, Reboleira et al. 2013a, Reboleira et al. 2013, Reboleira et al. 2015). With this research, we offer information about 15 cave-adapted isopods of continental Portugal, their distribution, habitats, species ecology, current threats and conservation measures needed. This information is fundamental to raise awareness on the threats that subterranean ecosystems and fauna face and to establish conservation measures to prevent their decline and possible extinction.

In the Iberian Peninsula, cave-adapted terrestrial isopods are also found in shallow subterranean habitats, such as the mesovoid shallow substrate (MSS) (Cifuentes and Barranco 2020). So far, previous biological investigations in the MSS in Portugal have only retrieved troglophile species of Oniscidea (Reboleira et al. 2015). Therefore, there is high potential for future findings of cave-adapted Oniscidea distributed also in the MSS, as recentely happened for other arthropod groups (Eusébio et al. 2021).

It is a priority to establish concrete protection strategies for cave-adapted species in continental Portugal. We need to improve the knowledge about population size and dynamics, real extent of subterranean distribution, improve our knowledge on the functional ecology, understand species life cycle and evaluate their sensitivity to disturbance. This contribution may be used as a support for decision-making for territory management and to define conservation measures for cave endemic species. Cave-adapted terrestrial isopods have the potential to be used as umbrella species for the conservation of other cave-adapted species sharing the same subterranean habitats.

Discussion

## Supplementary Material

2F1C3046-2169-5E36-AB53-F8D02E3A9E14 10.3897/BDJ.10.e78796.suppl1 Supplementary material 1 Distribution of cave-adapted terrestrial isopods in continental Portugal Data type Species distribution map Brief description (A) Overview of the distribution of cave-adapted terrestrial isopods in continental Portugal; (B) Sicó karst area distribution detail; (C) Estremenho and Montejunto karst massifs, Caldas da Rainha Typhonic Valley and Cesaredas Plateau distribution detail; (D) Algarve karst massif distribution detail; (E) Lisbon Peninsula and Arrábida karst massif and Estremoz-Cano karst massif distribution detail; and (F) Vimioso paleokarst distribution detail.
Species: *Trichoniscoidesbroteroi* (orange circle), *T.ouremensis* (pink circle), *T.serrai* (dark blue circle), *T.subterraneus* (yellow circle), *T.meridionalis* (purple circle), *T.bellesi* (forest green circle), *T.sicoensis* (light blue circle), *Metatrichoniscoidessalirensis* (dark blue diamond), *Troglonethesolissipoensis* (dark blue star), *T.arrabidaensis* (pink star), *Miktoniscuslongispina* (yellow cross), *Moseriusinexpectatus* (light blue hexagon), *Cordioniscuslusitanicus* (dark blue triangle outline), *Porcelliocavernicolus* (pink triangle) and *Troglelumamachadoi* (pink circle outline). File: oo_607434.png https://binary.pensoft.net/file/607434 A.S.P.S. Reboleira, R.P. Eusébio

DD91E314-7C4E-5774-8FF0-6086CE34BF8A 10.3897/BDJ.10.e78796.suppl2 Supplementary material 2 Distribution of *Trichoniscoidesbellesi* Data type Species distribution map Brief description *Trichoniscoidesbellesi* distribution: Algar do Javali Cave, Montejunto karst massif. File: oo_607707.tif https://binary.pensoft.net/file/607707 A.S.P.S. Reboleira, R.P.Eusébio

5914DE8F-D8BC-5B98-9A9F-9D4D7A6E1EE9 10.3897/BDJ.10.e78796.suppl3 Supplementary material 3 Distribution of *Trichoniscoidesbroteroi* Data type Species distribution map Brief description *Trichoniscoidesbroteroi* distribution: Alqueves Cave, Sicó karst area. File: oo_607708.tif https://binary.pensoft.net/file/607708 A.S.P.S. Reboleira, R.P. Eusébio

5FB36EF8-919B-5348-86EF-333B2BE58A35 10.3897/BDJ.10.e78796.suppl4 Supplementary material 4 Distribution of *Trichoniscoidesmeridionalis* Data type Species distribution map Brief description *Trichoniscoidesmeridionalis* distribution. (A) Detail of distribution: Algar do Vale do Pena; Alcobertas Cave; Algar do Zé de Braga Cave; Lapa da Chã de Cima Cave; Algar do Ladoeiro Cave; Algar do Pena Cave; Moinhos Velhos Cave; Papagaio Cave; Algar do Burro Cave; and Almonda Cave. File: oo_607711.tif https://binary.pensoft.net/file/607711 A.S.P.S. Reboleira, R.P. Eusébio

597F809C-3090-5016-9767-C3E76CCD0A16 10.3897/BDJ.10.e78796.suppl5 Supplementary material 5 Distribution of *Trichoniscoidesouremensis* Data type Species distribution map Brief description *Trichoniscoidesouremensis* distribution: Lapa da Salgada Cave, Fátima Plateau. File: oo_607712.tif https://binary.pensoft.net/file/607712 A.S.P.S. Reboleira, R.P. Eusébio

F92DCDAA-FA35-5326-85D4-F63245509A6C 10.3897/BDJ.10.e78796.suppl6 Supplementary material 6 Distribution of *Trichoniscoidesserrai* Data type Species distribution map Brief description *Trichoniscoidesserrai* distribution: Santo Adrião Cave, Vimioso karst area. File: oo_607713.tif https://binary.pensoft.net/file/607713 A.S.P.S. Reboleira, R.P. Eusébio

48CB91A0-5194-5E31-94A2-A512AA8133E5 10.3897/BDJ.10.e78796.suppl7 Supplementary material 7 Distribution of *Trichoniscoidessicoensis* Data type Species distribution map Brief description *Trichoniscoidessicoensis* distribution. (A) Detail of the distribution: Arrifana Cave; Santa Maria da Estrela Cave; Cerâmica Cave; São Simão Cave; Algarinho Cave; and Soprador do Carvalho Cave. File: oo_607714.tif https://binary.pensoft.net/file/607714 A.S.P.S. Reboleira, R.P.Eusébio

774C7847-3F7C-544E-81E9-48B9C9A29AFE 10.3897/BDJ.10.e78796.suppl8 Supplementary material 8 Distribution of *Trichoniscoidessubterraneus* Data type Species distribution map Brief description *Trichoniscoidessubterraneus* distribution: Alta do Cabeço dos Mosqueiros Cave, Aljubarrota Plateau. File: oo_607720.tif https://binary.pensoft.net/file/607720 A.S.P.S. Reboleira, R.P. Eusébio

71845157-2E1F-5082-AAA4-DDC0924AFDBD 10.3897/BDJ.10.e78796.suppl9 Supplementary material 9 Distribution of *Metatrichoniscoidessalirensis* Data type Species distribution map Brief description *Metatrichoniscoidessalirensis* distribution: Salir Cave, Caldas da Rainha. File: oo_607721.tif https://binary.pensoft.net/file/607721 A.S.P.S. Reboleira, R.P. Eusébio

425A22BF-FE18-5FDB-B23D-8937DEED57B6 10.3897/BDJ.10.e78796.suppl10 Supplementary material 10 Distribution of *Troglonethesolissipoensis* Data type Species distribution map Brief description *Troglonethesolissipoensis* distribution: Alvide Cave, Lisbon. File: oo_607722.tif https://binary.pensoft.net/file/607722 A.S.P.S. Reboleira, R.P. Eusébio

85C91C9B-5B83-533D-AB14-9F6ABE3F8A6B 10.3897/BDJ.10.e78796.suppl11 Supplementary material 11 Distribution of *Troglonethesarrabidaensis* Data type Species distribution map Brief description *Troglonethesarrabidaensis* distribution: Frade Cave, Arrábida karst massif. File: oo_607723.tif https://binary.pensoft.net/file/607723 A.S.P.S. Reboleira, R.P.Eusébio

BC5A499B-29AA-5FBE-B1CE-77CC7E4752A5 10.3897/BDJ.10.e78796.suppl12 Supplementary material 12 Distribution of *Miktoniscuslongispina* Data type Species distribution map Brief description *Miktoniscuslongispina* distribution. (A) Detail of distribution: Casal da Lebre Cave; Algar da Ervilha Cave; and Cerâmica Cave. File: oo_607725.tif https://binary.pensoft.net/file/607725 A.S.P.S. Reboleira, R.P. Eusébio

DB2A85A3-79E9-53CC-AC9E-E2C21922CF6F 10.3897/BDJ.10.e78796.suppl13 Supplementary material 13 Distribution of *Moseriusinexpectatus* Data type Species distribution map Brief description *Moseriusinexpectatus* distribution: Almonda Cave, Estremenho karst massif. File: oo_607726.tif https://binary.pensoft.net/file/607726 A.S.P.S. Reboleira, R.P. Eusébio

C9060AAB-01F8-5426-86EE-22A80F48CE21 10.3897/BDJ.10.e78796.suppl14 Supplementary material 14 Distribution of *Cordioniscuslusitanicus*

Data type Species distribution map Brief description *Cordioniscuslusitanicus* distribution. (A) Detail of distribution: Ibne Ammar Cave; Algarão do Remexido Cave; Vale Telheiro Cave; Senhora Cave; and Algar de Santo António Cave. File: oo_607729.tif https://binary.pensoft.net/file/607729 A.S.P.S. Reboleira, R.P. Eusébio

9A58FB28-25DF-5F94-842E-3D0690437F8C 10.3897/BDJ.10.e78796.suppl15 Supplementary material 15 Distribution of *Porcelliocavernicolus* Data type Species distribution map Brief description *Porcelliocavernicolus* distribution. (A) Detail of distribution: Gruta d’el Rey; Furjaca Cave; Santa Maria da Estrela Cave; Cerâmica Cave; Algarinho Cave; Soprador do Carvalho Cave; and Abrigo Tomar I Cave. File: oo_607732.tif https://binary.pensoft.net/file/607732 A.S.P.S. Reboleira, R.P. Eusébio

F77EA613-7319-514F-BCF6-CF2EFDC4F8E2 10.3897/BDJ.10.e78796.suppl16 Supplementary material 16 Distribution of *Troglelumamachadoi* Data type Species distribution map Brief description *Troglelumamachadoi* distribution. (A) Detail of distribution: Ibne Ammar Cave; Algarão do Remexido Cave; Vale Telheiro Cave; Algarão do Paulino Cave; Abismo Novo Cave; and Senhora Cave. File: oo_607736.tif https://binary.pensoft.net/file/607736 A.S.P.S. Reboleira, R.P. Eusébio

## References

[B7528380] Campos-Filho Ivanklin Soares, Araujo Paula Beatriz, Bichuette Maria Elina, Trajano Eleonora, Taiti Stefano (2014). Terrestrial isopods (Crustacea: Isopoda: Oniscidea) from brazilian caves. Zoological Journal of the Linnean Society.

[B7347906] Castaño-Sánchez Andrea, Hose Grant C., Reboleira Ana Sofia P. S. (2020). Ecotoxicological effects of anthropogenic stressors in subterranean organisms: a review. Chemosphere:.

[B7534170] Cifuentes J., Barranco P. (2020). *Porcellioselomai* sp. n. (Oniscidea, Porcellionidae), un nuevo isópodo terrestre del medio subterráneo superficial (MSS) de la Península Ibérica. Boletín de la Asociación Española de Entomología.

[B7346978] Cruz A. (1993). Especies nuevas o poco conocidas de isopodos terrestres de la Península Ibérica. III. *Trichoniscoidespitarquensis* sp. n. y *T.serrai* sp. n. (Crustacea, Oniscidea, Trichoniscidae).. Bulletin de la Société d’Histoire Aturelle de Toulouse.

[B7033455] Directive H. (1992). Council directive 92/43/EEC of 21 May 1992 on the conservation of natural habitats and of wild fauna and flora.. Official Journal of the European Union.

[B7538831] Enghoff H., Reboleira A. S.P.S. (2013). Subterranean species of *Acipes* Attems, 1937 (Diplopoda, Julida, Blaniulidae). Zootaxa:.

[B7534179] Eusébio R. P., Enghoff H., Solodovnikov A., Michelsen A., Barranco P., Salgado J. M., Sendra A., Reboleira A. S.P.S. (2021). Temporal and spatial dynamics of arthropod groups in terrestrial subsurface habitats in central Portugal. Zoology:.

[B7004184] Grilo M. J., Grilo F., Silva M., Lopes L. Sistema de informação para o património arquitetónico. Gruta dos Alqueves / cova da Moura. http://www.monumentos.gov.pt/Site/APP_PagesUser/SIPA.aspx?id=2652.

[B7528296] Hassall M., Turner J. G., Rands M. R. W. (1987). Effects of terrestrial isopods on the decomposition of woodland leaf litter. Oecologia.

[B7032562] Hoffmann D. L., Pike A. W.G., Wainer K., Zilhão J. (2013). New u-series results for the speleogenesis and the palaeolithic archaeology of the Almonda karstic system (Torres Novas, Portugal). Quaternary International.

[B7528261] Hornung Elisabeth (2011). Evolutionary adaptation of oniscidean isopods to terrestrial life: structure, physiology and behavior. Terrestrial Arthropod Reviews.

[B7033464] ICNB (2000). Grutas não exploradas pelo turismo.. http://www2.icnf.pt/portal/pn/biodiversidade/rn2000/resource/doc/rn-plan-set/hab/hab-8310/view.

[B7075146] ICNF Rede nacional de áreas protegidas (RNAP). https://sig.icnf.pt/portal/home/item.html?id=02b7a03f8fbd4dada77f5f3e5f91f186.

[B7534193] Mammola Stefano, Cardoso Pedro, Culver David C, Deharveng Louis, Ferreira Rodrigo L, Fišer Cene, Galassi Diana M P, Griebler Christian, Halse Stuart, Humphreys William F, Isaia Marco, Malard Florian, Martinez Alejandro, Moldovan Oana T, Niemiller Matthew L, Pavlek Martina, Reboleira Ana Sofia P S, Souza-Silva Marconi, Teeling Emma C, Wynne J Judson, Zagmajster Maja (2019). Scientists' warning on the conservation of subterranean ecosystems. BioScience.

[B7346987] Moreira D., Moreira P. (2006). Minas de Santo Adrião I, minas de Santo Adrião II, gruta do Dique, mina de Água, gruta grande. Espeleo Divulgação.

[B7534161] Neves J., Pessoa M., Redinha N. (2005). O sistema espeleológico do Dueça. Espeleo Divulgação.

[B7033484] Nóbrega A., Carvalho F., Alte da Veiga F., Soares M., Neves J., Pupo Correia J. (1984). Gruta da Cerâmica.. Espeleo Divulgação.

[B7052039] Piccini L., Di Lorenzo T., Costagliola P., Galassi D. M.P. (2019). Marble slurry’s impact on groundwater: the case study of the Apuan Alps karst aquifers. Water.

[B7528187] Ravn Nynne Rand, Michelsen Anders, Reboleira Ana Sofia P. S. (2020). Decomposition of organic matter in caves. Frontiers in Ecology and Evolution.

[B7528287] Reboleira Ana, Gonçalves Fernando, Oromí Pedro (2013). Literature survey, bibliographic analysis and a taxonomic catalogue of subterranean fauna from Portugal. Subterranean Biology.

[B7032571] Reboleira A. S.P.S. (2007). Os coleópteros (Insecta, Coleoptera) cavernícolas do Maciço Calcário Estremenho: uma aproximação à sua biodiversidade..

[B7032580] Reboleira A. S.P.S., Gonçalves F. J., Serrano A. R.M. (2009). Two new species of cave dwelling *Trechus* Clairville, 1806 of the *fulvus* - group (Coleoptera, Carabidae, Trechinae) from Portugal. Deutsche Entomologische Zeitschrift.

[B7551036] Reboleira A. S., Ortuño V. M., Gonçalves F., Oromí P. (2010). A hypogean new species of *Trechus* Clairville, 1806 (Coleoptera, Carabidae) from Portugal and considerations about the *T.fulvus* - species group. Zootaxa.

[B7538785] Reboleira Ana Sofia P. S., Zaragoza J., Gonçalves F., Oromí P. (2010). *Titanobochica*, surprising discovery of a new cave-dwelling genus from southern Portugal (Arachnida: Pseudoscorpiones: Bochicidae). Zootaxa.

[B7052029] Reboleira A. S.P.S., Borges P., Gonçalves F., Serrano A., Oromí P. (2011). The subterranean fauna of a biodiversity hotspot region - Portugal: an overview and its conservation. International Journal of Speleology.

[B7075122] Reboleira A. S.P.S. (2012). Biodiversity and conservation of subterranean fauna of Portuguese karst..

[B7538880] Reboleira A. S.P.S., Gonçalves F., Oromí P., Mendes L. (2012). *Squamatiniaalgharbica* gen. n. sp. n., a remarkable new Coletiniinae silverfish (Zygentoma: Nicoletiidae) from caves in southern Portugal. Zootaxa.

[B7538803] Reboleira A. S.P.S., Zaragoza J., Gonçalves F., Oromí P. (2012). *Lusoblothrus*, a new syarinid pseudoscorpion genus (Arachnida) from Portugal, cccupying an isolated position within the holarctic fauna. Zootaxa.

[B7538822] Reboleira A. S.P.S., Enghoff H. (2013). The genus *Boreviulisoma* Brolemann, 1928 - an Iberian-N African outlier of a mainly tropical tribe of millipedes (Diplopoda: Polydesmida: Paradoxosomatidae). Zootaxa.

[B7347915] Reboleira Ana Sofia P. S., Abrantes Nelson, Oromí Pedro, Gonçalves Fernando (2013). Acute toxicity of copper sulfate and potassium dichromate on stygobiont *Proasellus*: general aspects of groundwater ecotoxicology and future perspectives. Water, Air, & Soil Pollution.

[B7350753] Reboleira Ana Sofia P. S., Zaragoza Juan A., Gonçalves Fernando, Oromí Pedro (2013). On hypogean *Roncocreagris* (Arachnida: Pseudoscorpiones: Neobisiidae) from Portugal, with descriptions of three new species. Zootaxa.

[B7003988] Reboleira A. S.P.S., Gonçalves F., Oromí P., Taiti S. (2015). The cavernicolous Oniscidea (Crustacea: Isopoda) of Portugal.. European Journal of Taxonomy.

[B7538862] Reboleira Ana Sofia P. S., Fresnada Javier, Salgado José Maria (2017). A new species of *Speonemadus* from Portugal, with the revision of the *escalerai* - group (Coleoptera, Leiodidae). European Journal of Taxonomy.

[B7349009] Reboleira Ana Sofia P. S., Enghoff Henrik (2018). First continental troglobiont *Cylindroiulus* millipede (Diplopoda, Julida, Julidae). ZooKeys.

[B7538841] Reboleira A. S.P.S., Sendra A., Gonçalves F., Oromí P. (2019). The first hypogean dipluran from Portugal: description of a new species of the genus *Litocampa* (Diplura: Campodeidae). Zootaxa.

[B7528313] Reboleira Ana Sofia, Eusébio Rita (2021). Cave-adapted beetles from continental Portugal. Biodiversity Data Journal.

[B7538905] Ribera C. (1993). *Dysderacaeca* n. sp. y *Harpacteastalitoides* n. sp. (Araneae), dos especies cavernícolas de Marruecos y Portugal. Revue Arachnologique.

[B7052020] Ribera I., Reboleira A. S.P.S. (2019). The first stygobiont species of Coleoptera from Portugal, with a molecular phylogeny of the s*iettitia* - group of genera (Dytiscidae, Hydroporinae, Hydroporini, Siettitiina). ZooKeys.

[B7032591] Thomas C. (1991). Gruta da nascente d'Almonda. SIFON Bulletin Interne des Commissions Plongée Souterraine de l’Ile de France.

[B7006502] Vandel A. (1946). Crustacés isopodes terrestres (Oniscoïdea) épigés et cavernicoles du Portugal..

[B7528325] van Gestel Cornelis A. M., Loureiro Susana, Zidar Promoz (2018). Terrestrial isopods as model organisms in soil ecotoxicology: a review. ZooKeys.

[B7350771] Zaragoza Juan A., Reboleira Ana Sofia P. S. (2018). Five new hypogean *Occidenchthonius* (Pseudoscorpiones: Chthoniidae) from Portugal. Journal of Arachnology.

