# Supplementary figures and images for: Species conservation profiles of cave-adapted terrestrial isopods from Portugal
Source: Biodivers Data J. 2022 Feb 28;10:e78796. doi: 10.3897/BDJ.10.e78796 (PMC8901612; doi:10.3897/BDJ.10.e78796)

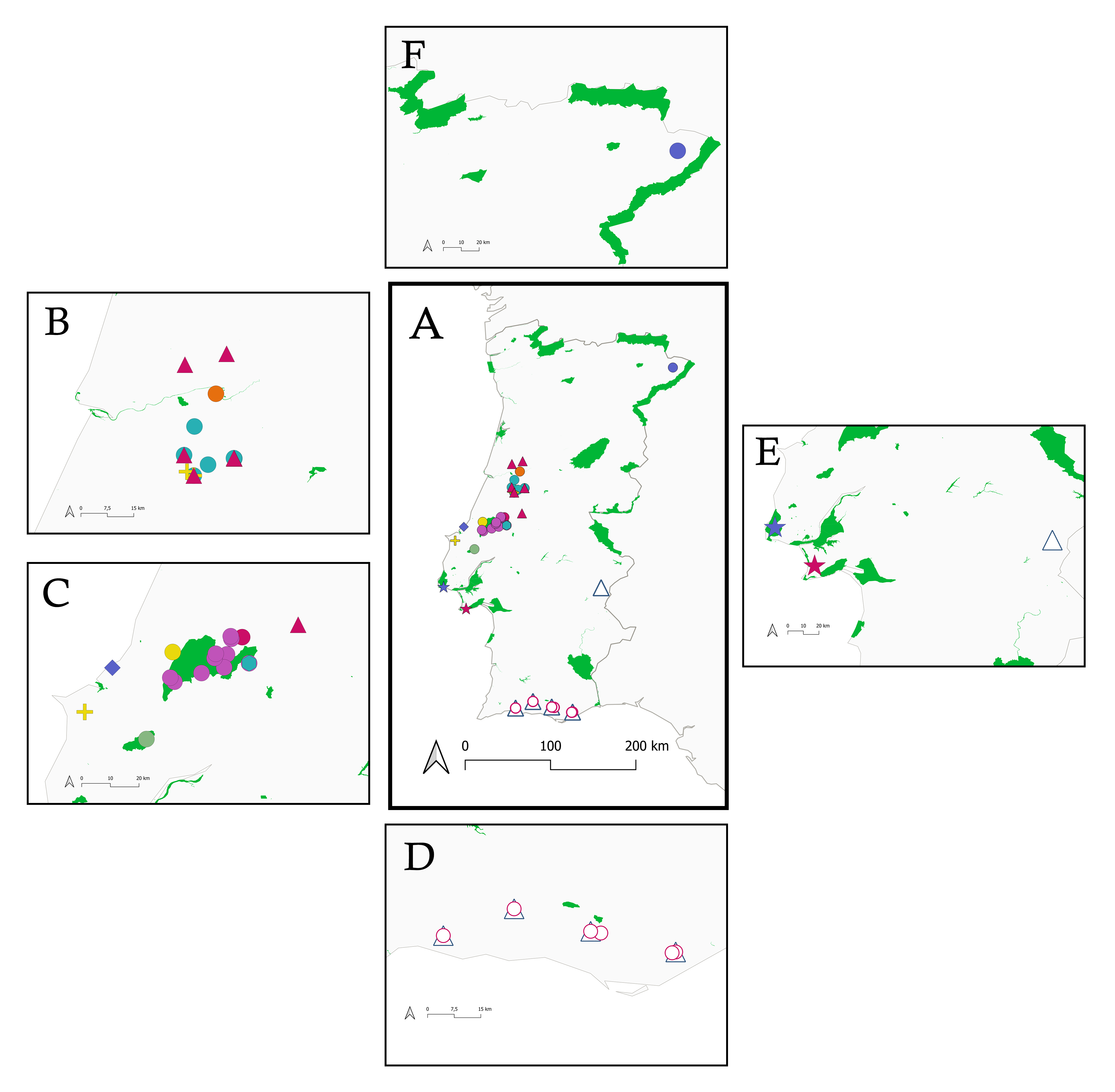

Supplement: Supplementary material 1 — Distribution of cave-adapted terrestrial isopods in continental Portugal [file bdj-10-e78796-s001.png]

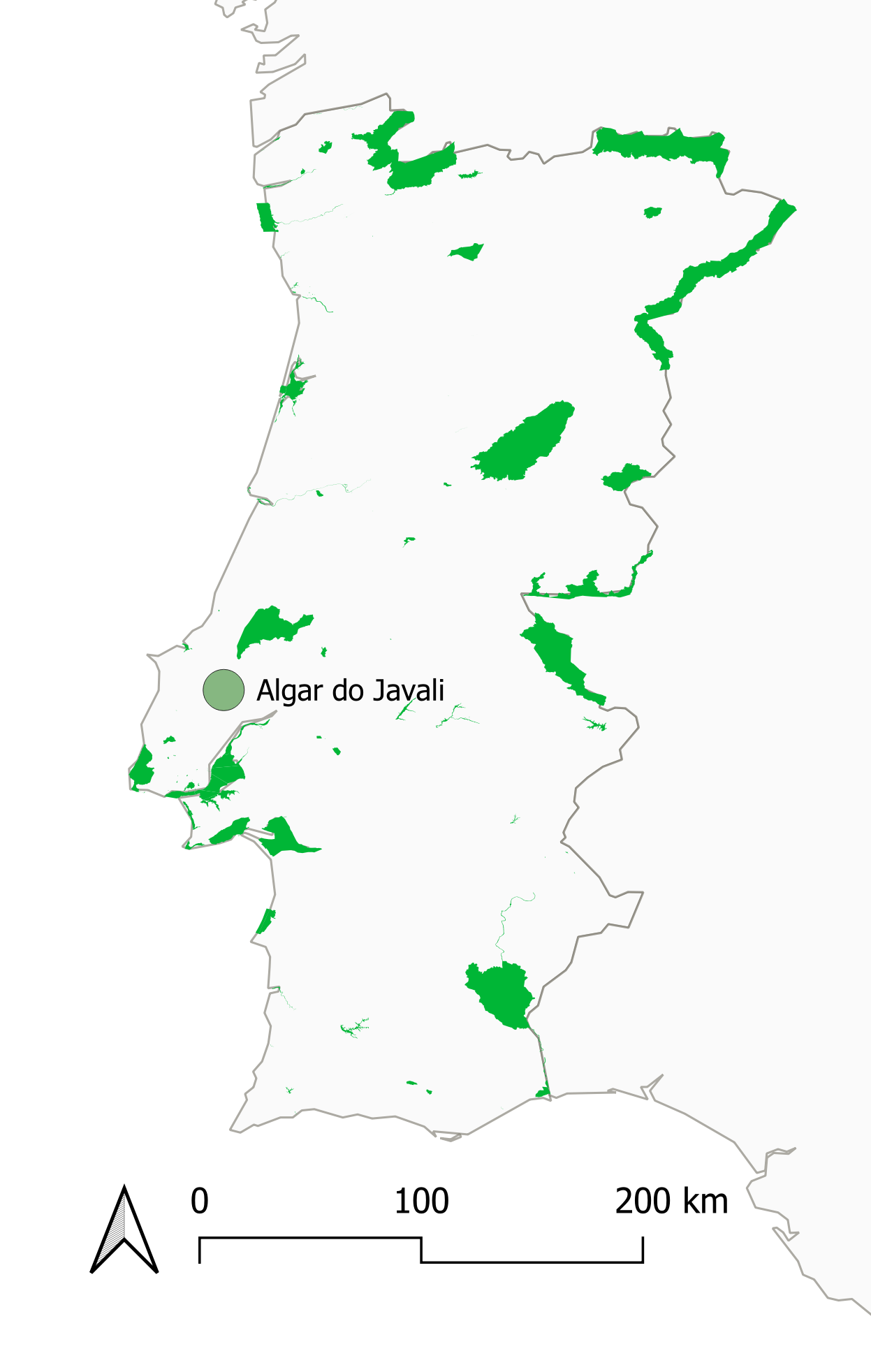

Supplement: Supplementary material 2 — Distribution of Trichoniscoidesbellesi [file bdj-10-e78796-s002.tif]

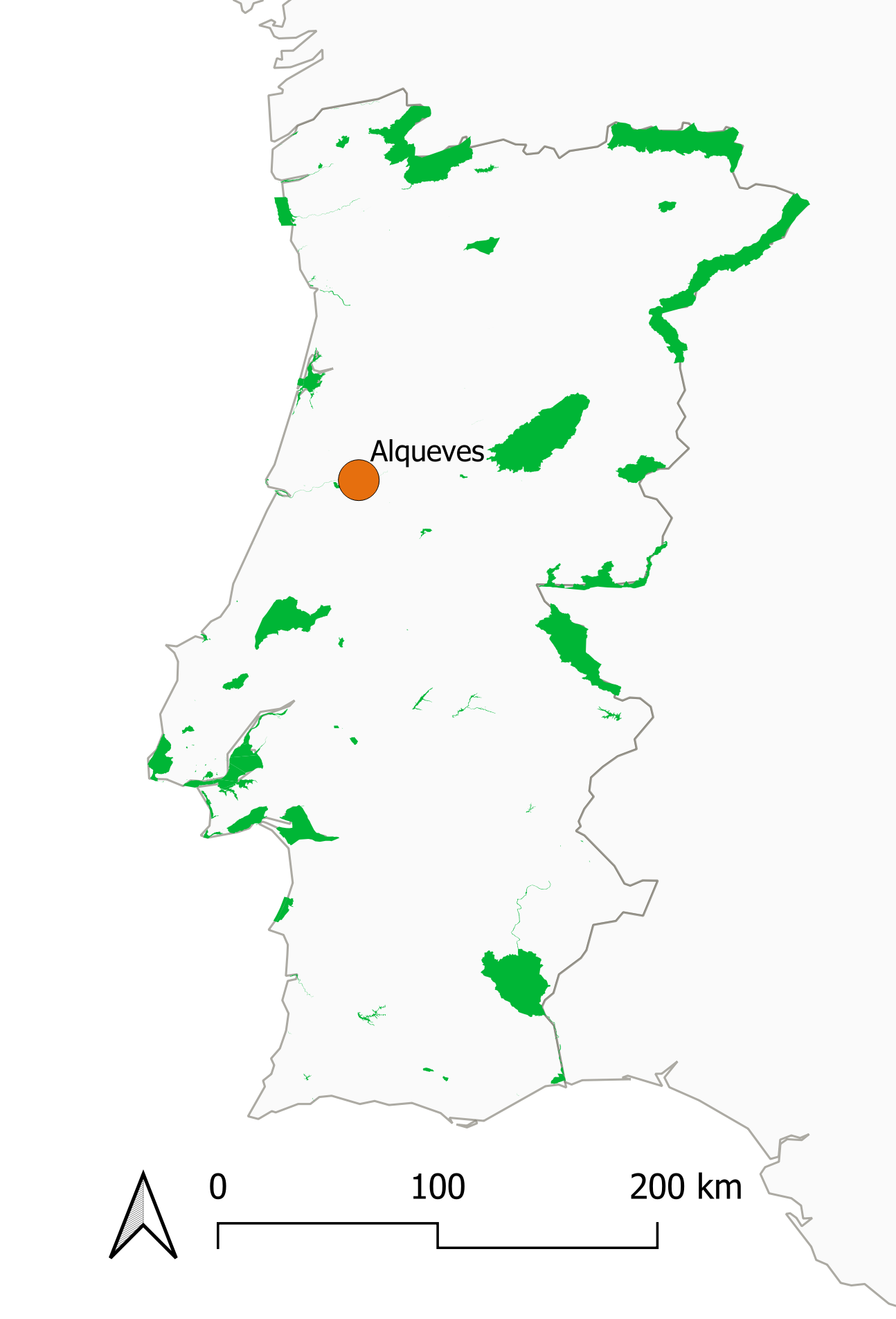

Supplement: Supplementary material 3 — Distribution of Trichoniscoidesbroteroi [file bdj-10-e78796-s003.tif]

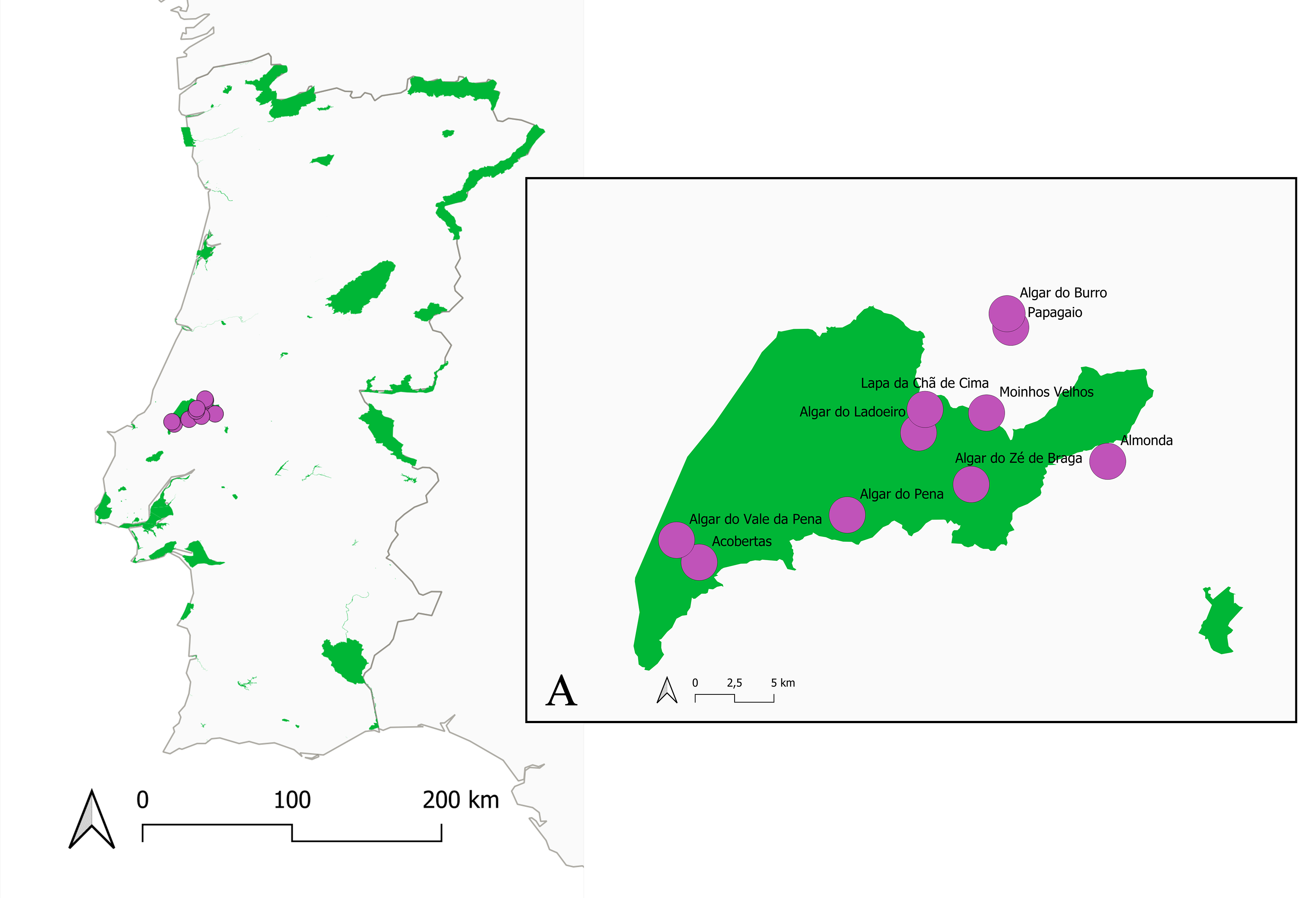

Supplement: Supplementary material 4 — Distribution of Trichoniscoidesmeridionalis [file bdj-10-e78796-s004.tif]

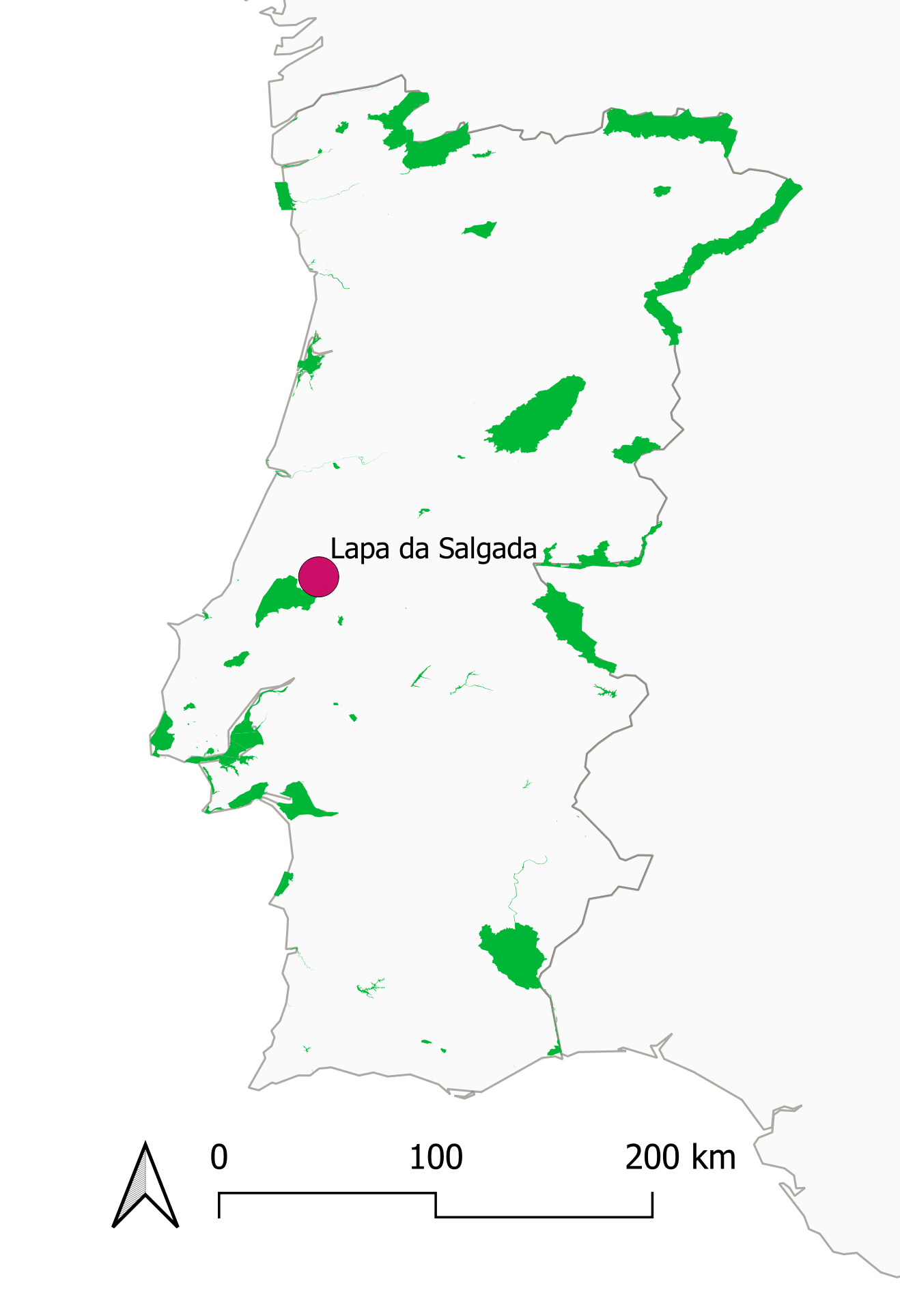

Supplement: Supplementary material 5 — Distribution of Trichoniscoidesouremensis [file bdj-10-e78796-s005.tif]

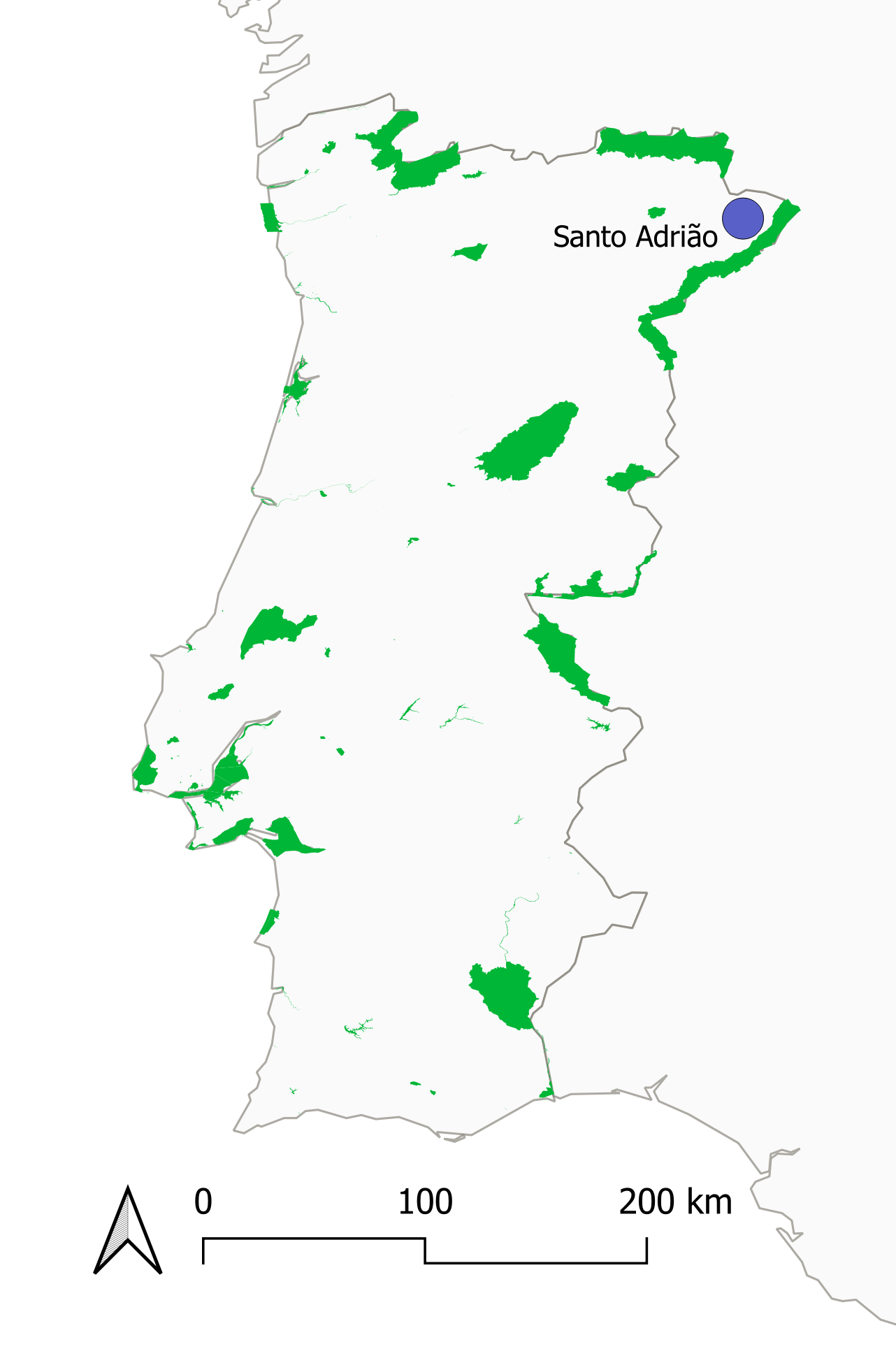

Supplement: Supplementary material 6 — Distribution of Trichoniscoidesserrai [file bdj-10-e78796-s006.tif]

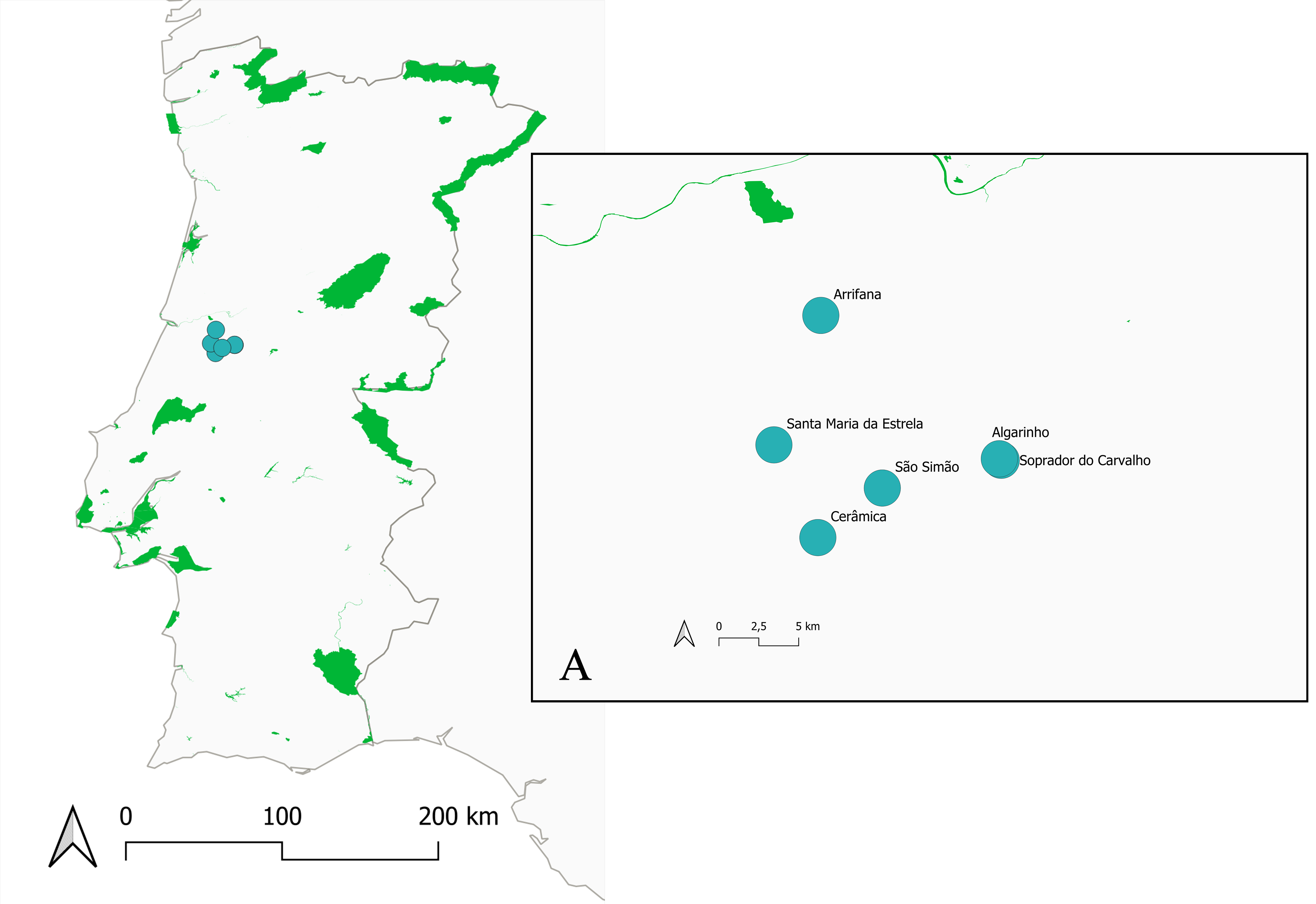

Supplement: Supplementary material 7 — Distribution of Trichoniscoidessicoensis [file bdj-10-e78796-s007.tif]

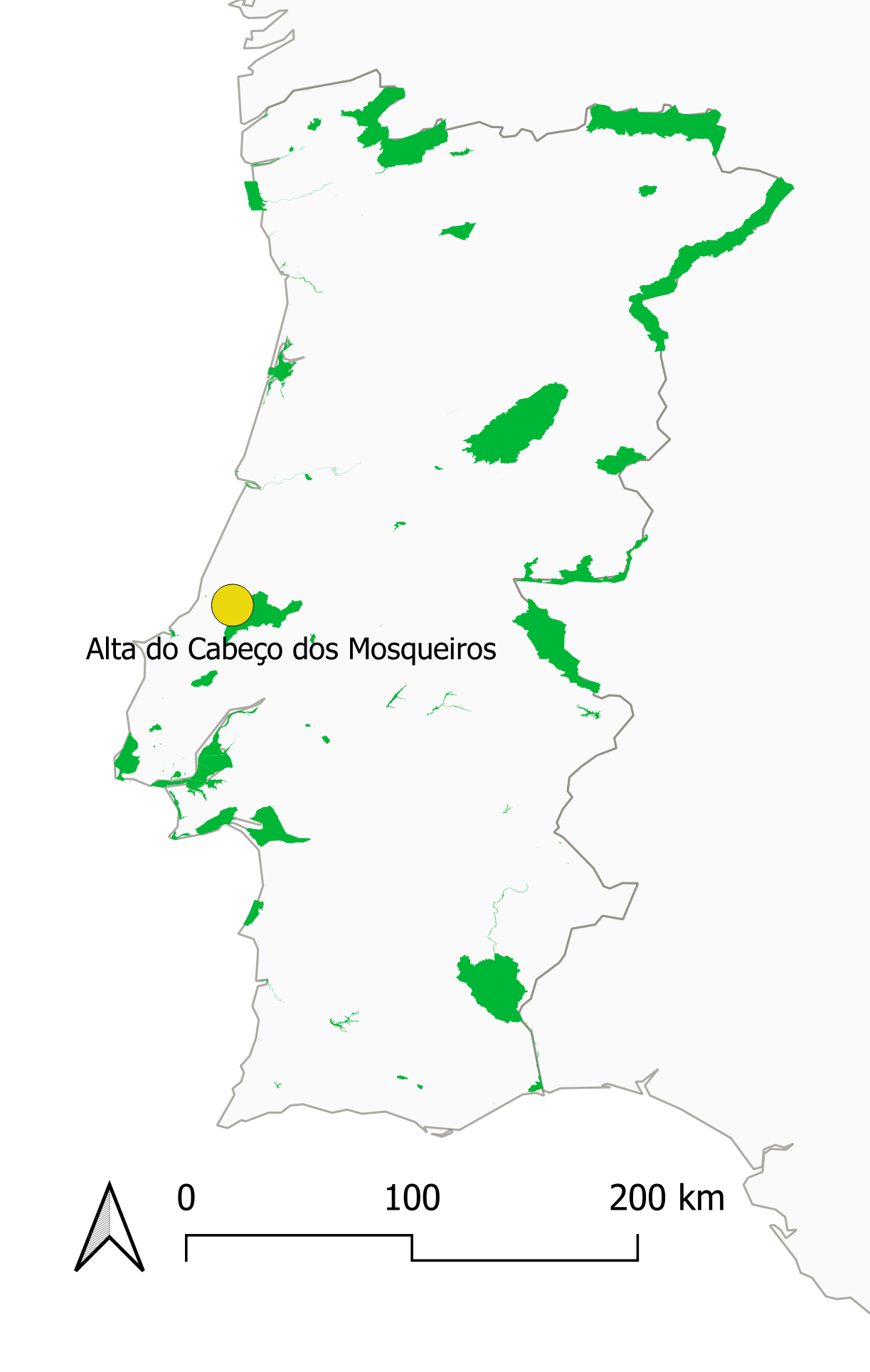

Supplement: Supplementary material 8 — Distribution of Trichoniscoidessubterraneus [file bdj-10-e78796-s008.tif]

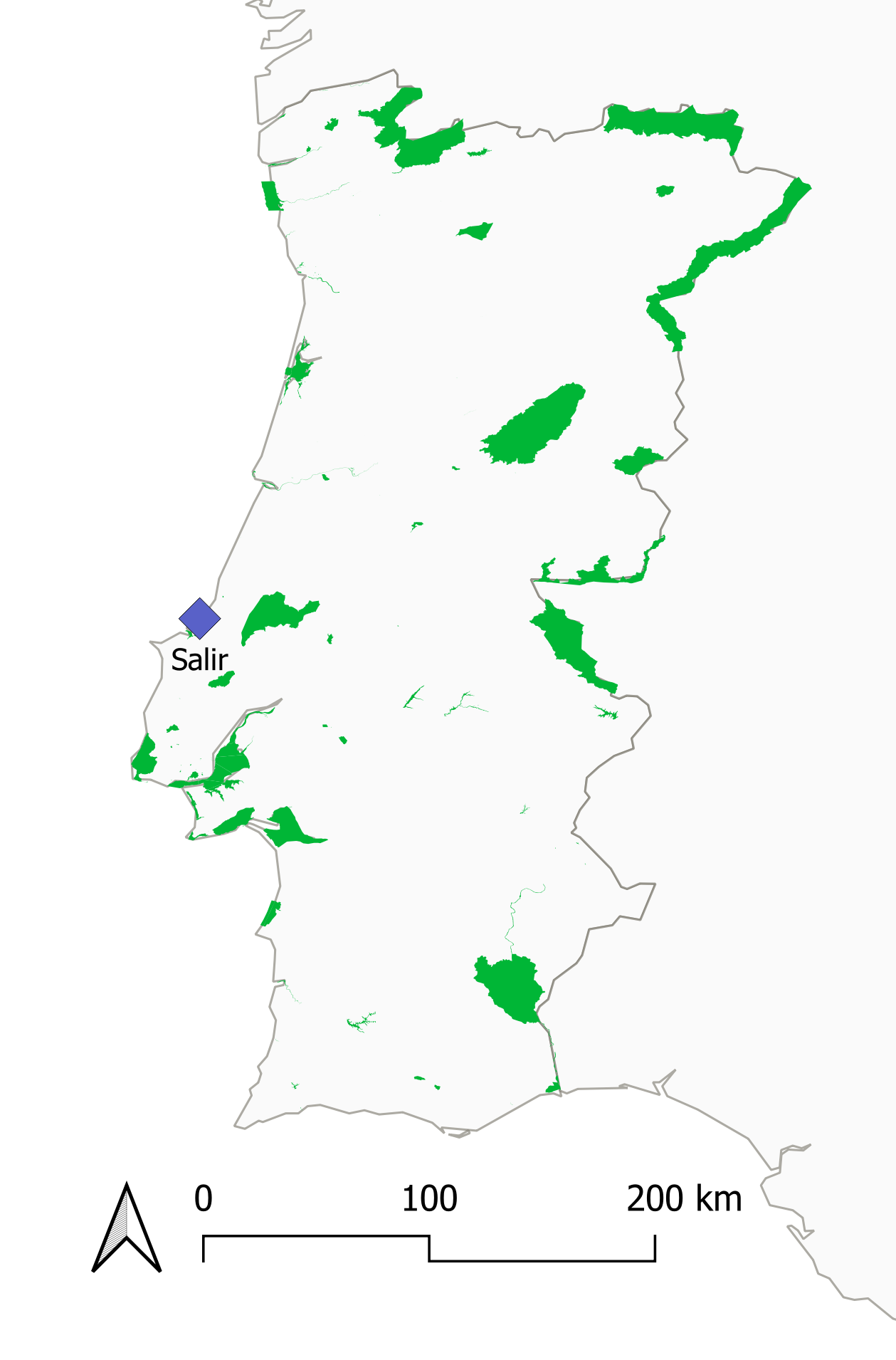

Supplement: Supplementary material 9 — Distribution of Metatrichoniscoidessalirensis [file bdj-10-e78796-s009.tif]

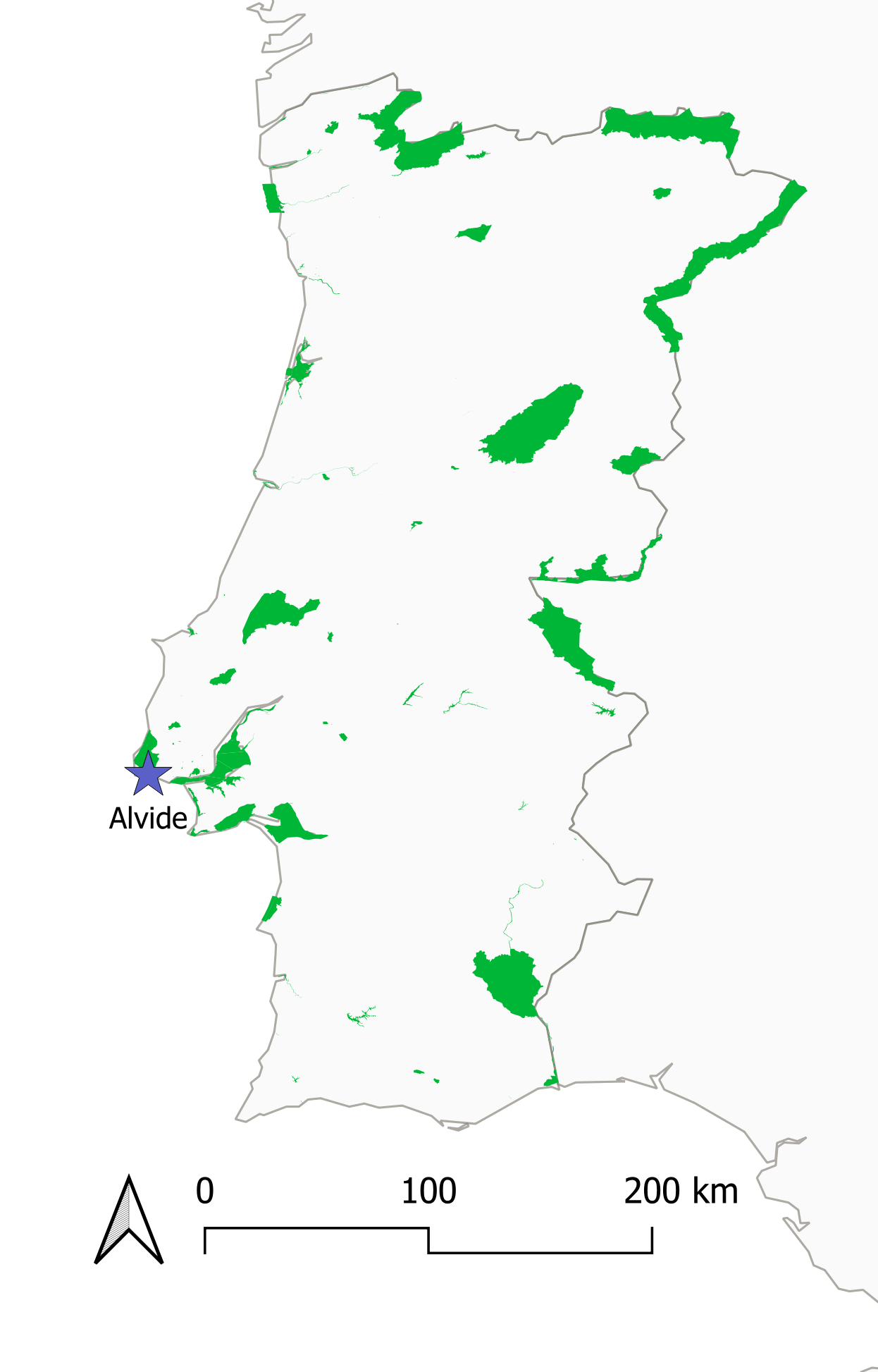

Supplement: Supplementary material 10 — Distribution of Troglonethesolissipoensis [file bdj-10-e78796-s010.tif]

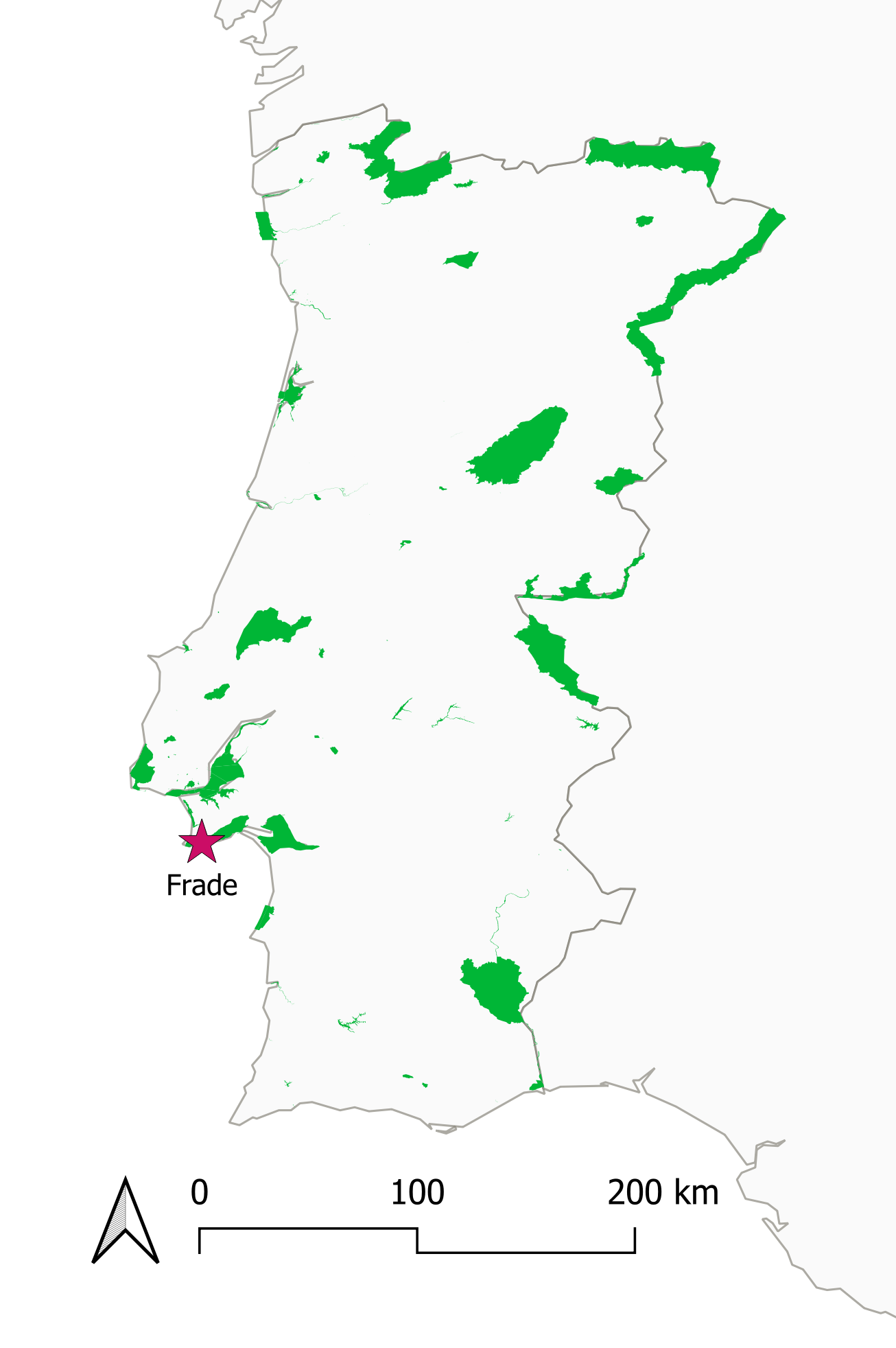

Supplement: Supplementary material 11 — Distribution of Troglonethesarrabidaensis [file bdj-10-e78796-s011.tif]

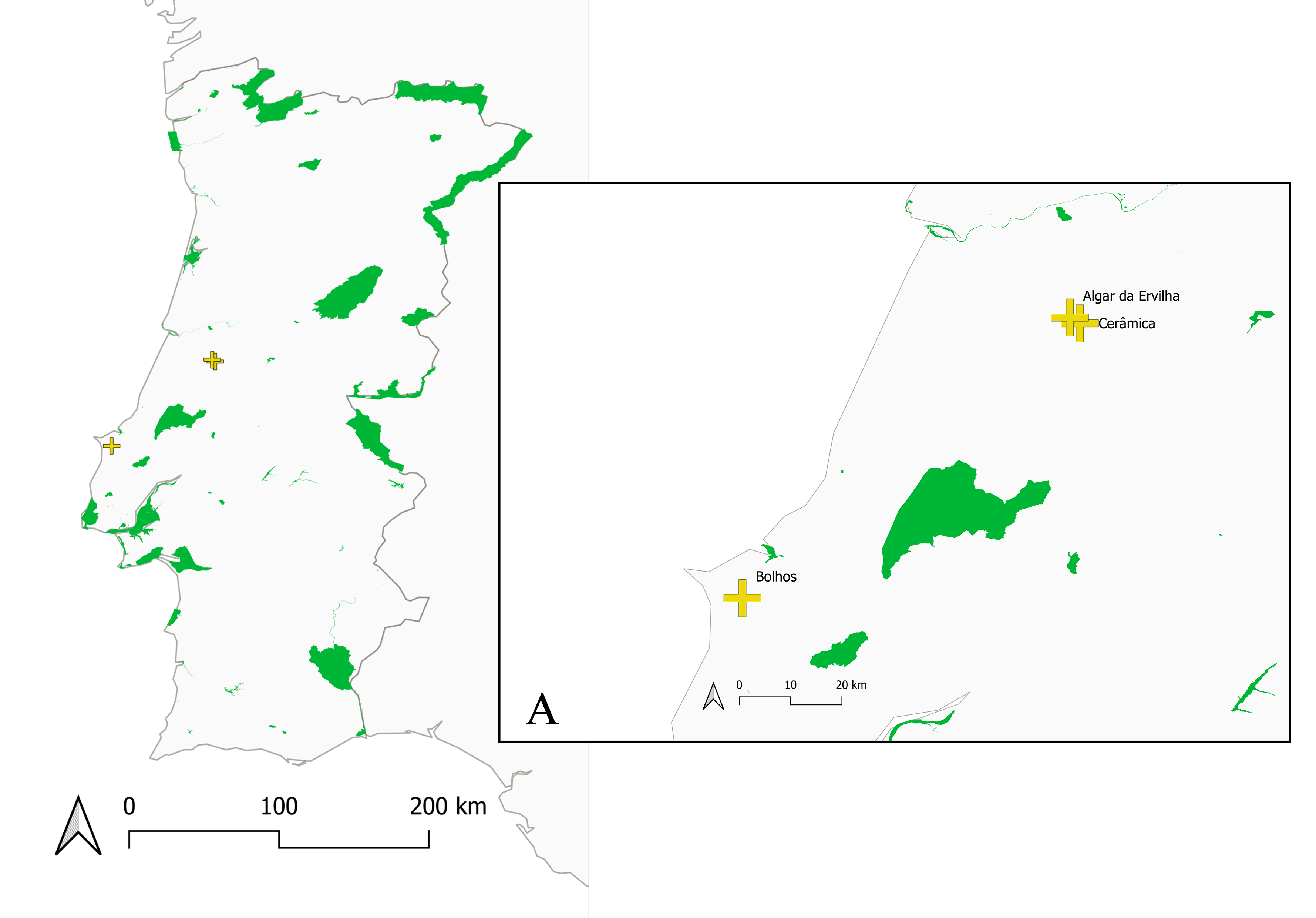

Supplement: Supplementary material 12 — Distribution of Miktoniscuslongispina [file bdj-10-e78796-s012.tif]

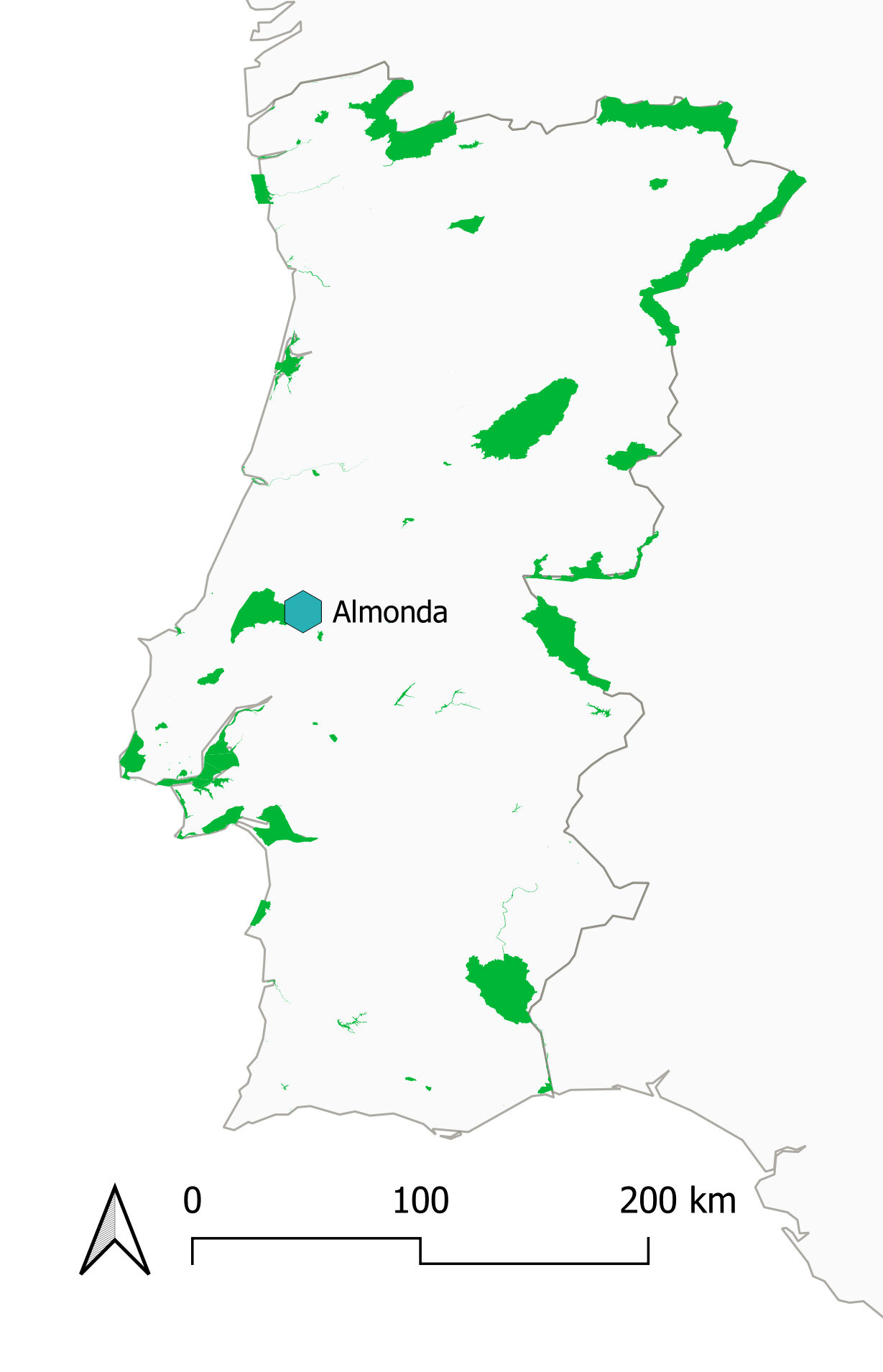

Supplement: Supplementary material 13 — Distribution of Moseriusinexpectatus [file bdj-10-e78796-s013.tif]

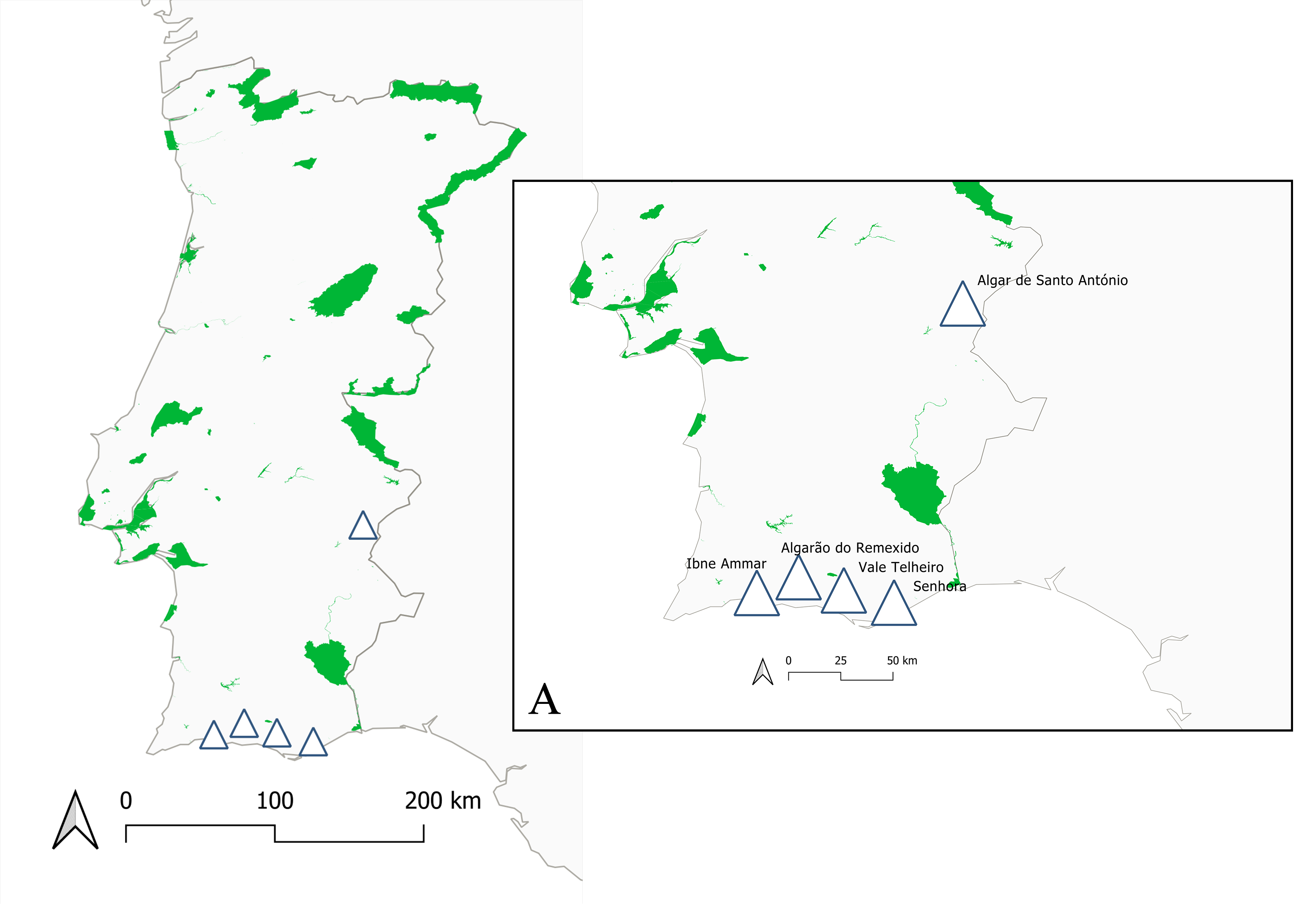

Supplement: Supplementary material 14 — Distribution of Cordioniscuslusitanicus [file bdj-10-e78796-s014.tif]

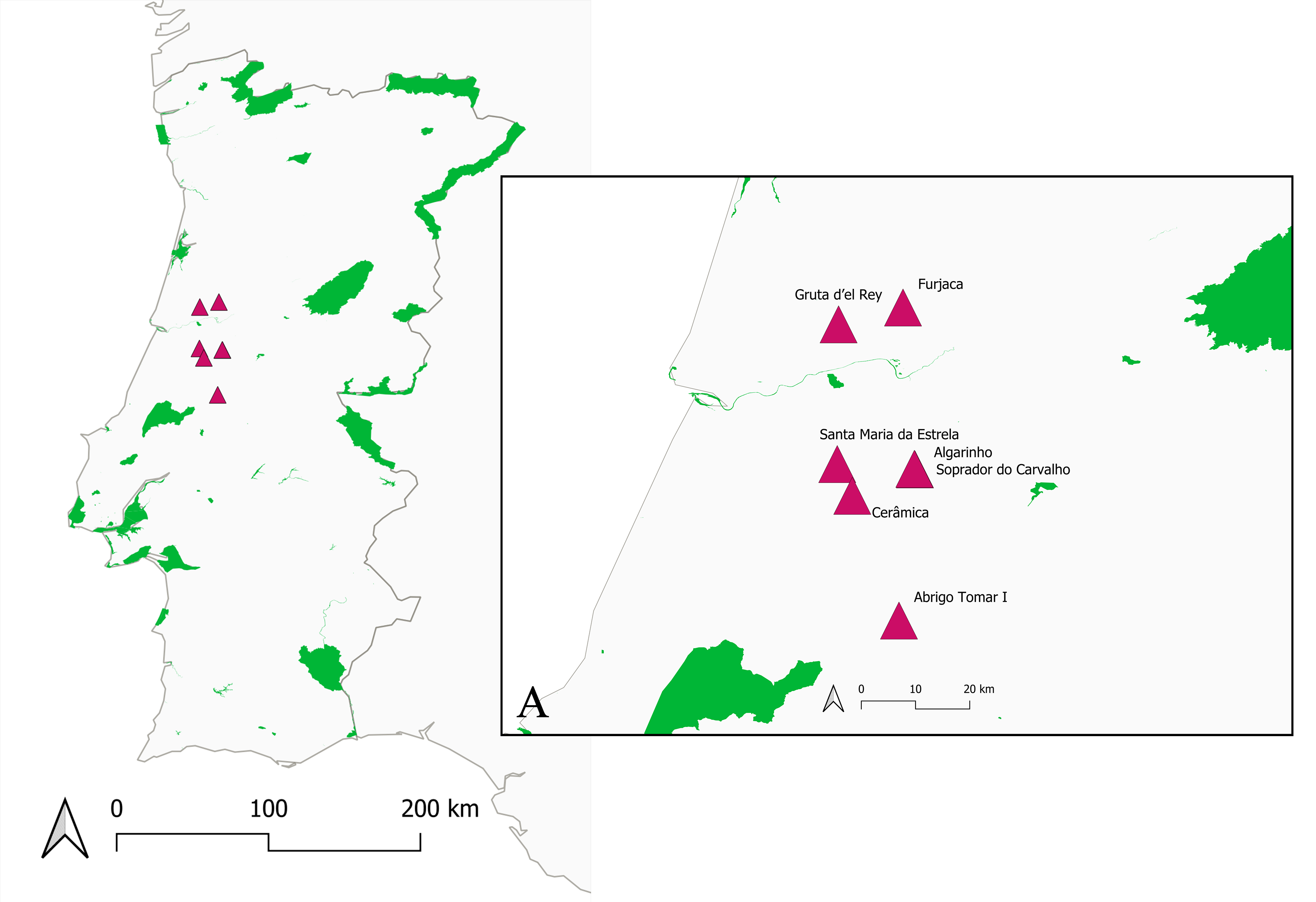

Supplement: Supplementary material 15 — Distribution of Porcelliocavernicolus [file bdj-10-e78796-s015.tif]

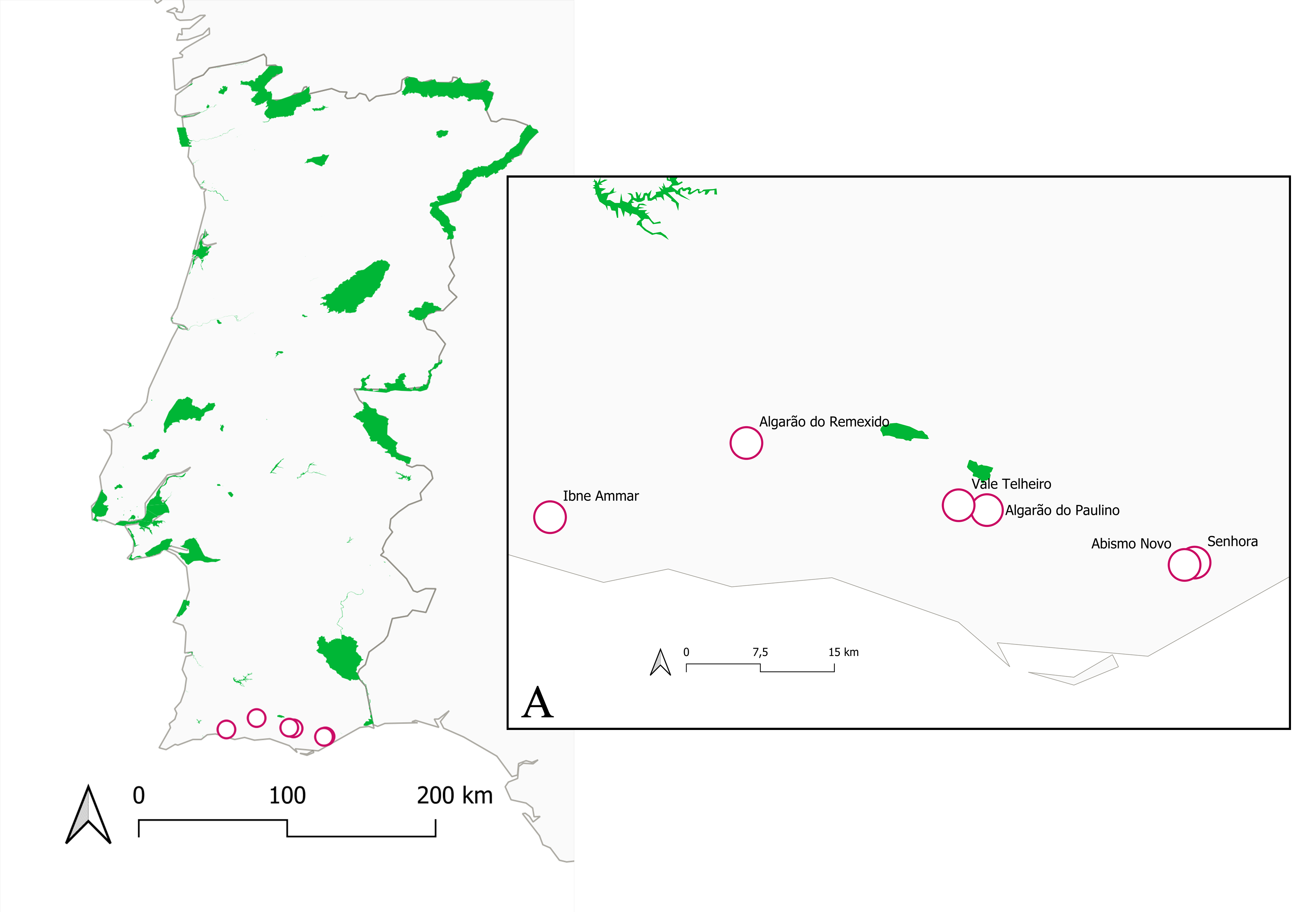

Supplement: Supplementary material 16 — Distribution of Troglelumamachadoi [file bdj-10-e78796-s016.tif]
